# TikTok is a valuable data source for tracking the opioid crisis

**DOI:** 10.1038/s41746-026-02654-x

**Published:** 2026-04-27

**Authors:** Issah A. Samori, Kristy A. Carpenter, Delaney A. Smith, Keith Humphreys, Anna Lembke, Johannes C. Eichstaedt, Russ B. Altman

**Affiliations:** 1 Department of Bioengineering, Stanford University, Stanford, CA USA; 2 Department of Biomedical Data Science, Stanford University, Stanford, CA USA; 3 Department of Biochemistry, Stanford University, Stanford, CA USA; 4 Department of Psychiatry and Behavioral Sciences, Stanford University, Stanford, CA USA; 5 Veterans Affairs Health Care System, Palo Alto, CA USA; 6 Department of Psychology, Stanford University, Stanford, CA USA; 7 Institute for Human-Centered AI, Stanford University, Stanford, CA USA; 8 Decision Sciences, INSEAD, Fontainebleau, France; 9 Department of Genetics, Stanford University, Stanford, CA USA; 10 Department of Medicine, Stanford University, Stanford, CA USA

**Keywords:** Diseases, Health care, Medical research

## Abstract

Monitoring opioid-related chatter on social media can predict the course of opioid addiction and the overdose epidemic. We assessed the utility of TikTok, a prominent short video-based social media platform, as a means of tracking the opioid addiction and overdose crisis. We collected 569,581 TikTok comments (posted between January 2021 and June 2025) from 48,306 opioid-related videos, making this study the first large-scale analysis of TikTok comments for opioid surveillance. We extracted 200 topics from these comments using Latent Dirichlet Allocation (LDA) and incorporated the topics into ARIMA models that forecast synthetic opioid mortality over 6-month horizons. We also analyzed conversational patterns using the LIWC2015 pronoun dictionaries and GPT o1-mini. We found that (1) incorporating TikTok topics into the ARIMA models reduced forecasting Mean Absolute Error by up to 37% (2) the topics spanned five broad themes (use, source, recovery, harm-reduction, loss), showing the diversity of opioid discourse on TikTok, and (3) TikTok comments included first-person, second-person, and third-person accounts of opioid use (i.e., personal use, engaging with other users in conversation about their use, and relating views of others’ use, respectively). These findings emphasize the usefulness of TikTok comments as a data source for opioid use surveillance.

## Introduction

Opioids are useful in pain management but carry the risk of addiction and overdose^1^. The increased availability and prescription of legally produced opioids beginning in the mid-1990s, an influx of heroin in the late 2000s, and the emergence of illicitly-manufactured synthetic opioids, such as fentanyl, in the 2010s have led to the widespread misuse of and addiction to opioids, creating an overdose epidemic^2,3^. This opioid crisis has been devastating, particularly in North America, claiming over 79,030 lives in the US in 2024 alone—over 50% more than the number of deaths caused by firearms^4,5^. According to the White House, in 2023, the opioid epidemic cost the US about $2.7 trillion in terms of healthcare costs and loss of quality of life^6^. To respond to such challenges, accurate and timely data on the status and future course of the epidemic is essential.

The U.S. Centers for Disease Control and Prevention’s (CDC) official reporting on overdose deaths lags by at least 6 months^7^. This lack of timely data makes it difficult for policymakers to effectively deploy resources, in turn making this crisis difficult to combat^2,7^. Prior studies have demonstrated the use of opioid term mentions on social media to provide almost real-time estimates of overdose deaths^8–19^. Many of these studies rely on Reddit or Twitter (now known as X). In recent years, short-form video platforms have exploded in popularity, especially among young adults, and a prominent example is TikTok^20^. Furthermore, recent changes to data access policy severely limit access to data on Reddit and Twitter^21^ and threaten the long-term utility of these platforms for monitoring the opioid crisis.

The TikTok platform has rapidly grown in popularity with over 150 million American users (almost half of the US population) as of March 2023^22^. TikTok enables users to create and share short-form videos, typically lasting between 15 and 60 s. Video creators can write a brief caption for their video, which may optionally include hashtagged keywords. Other users can engage with these videos through likes, comments, and shares. Our previous review on all social media platforms for opioid monitoring identified TikTok as a promising tool for tracking the opioid epidemic^21^. It also has an Application Programming Interface (API) that is freely available to researchers for data access.

Several studies have leveraged TikTok as a data source to examine substance use disorder (SUD) related content. Boling et al. analyzed 100 TikTok videos to characterize TikTok discussions around the overdose rescue medication, as well as harm reduction in general^23^. Sun et al. investigated how e-cigarettes are portrayed on TikTok^24^. Russell et al. characterized how people use TikTok while in recovery from SUD^25^. In addition to the videos themselves, the TikTok comments section also contains extensive discussions, but no study has used the rich comment data available on TikTok as a means of tracking the opioid addiction and overdose crisis and predicting the trajectory of the crisis in real time.

Accordingly, this study asked two questions: (i) What kinds of opioid-related texts are present in TikTok comments? (ii) Can the analysis of these texts improve overdose death prediction? To answer question (i), we characterized the TikTok comments using two different approaches. Firstly, we analyzed the comments to find what themes of opioid chatter are present on TikTok. Secondly, we analyzed the perspective with which these comments were authored to better understand the context of these opioid themes. Our analysis reveals that TikTok comments contain opioid-related discussions encompassing five major opioid-related themes: use of opioids, source of opioid acquisition, recovery from OUD, harm reduction, and loss of lives to opioids. We also found that TikTok hosts opioid use narratives from all three points of view (POVs), suggesting that TikTok users talk about their personal experiences with opioids, as well as other people’s experiences. Lastly, we showed that the volume of opioid comments on TikTok is predictive of real-world opioid overdose deaths.

## Results

### TikTok comments dataset summary statistics

**Fig. 1:**
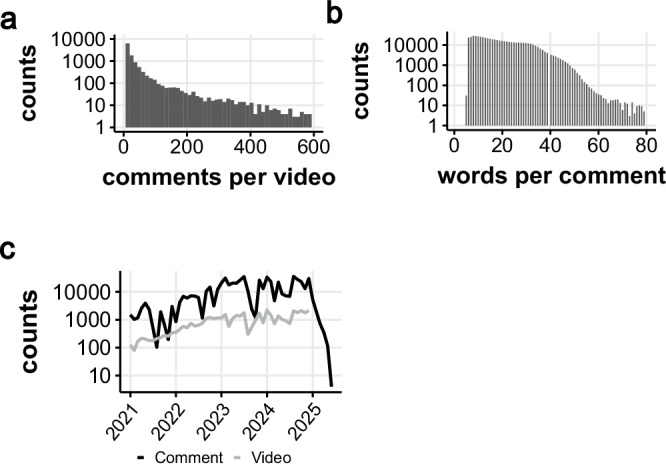
TikTok data descriptors. **a** Distribution of comment count per video. Mean = 15 comments and Standard deviation = 72 comments. **b** Distribution of comment lengths measured in number of words (1-gram tokens). Mean comment length is 20 words, and the standard deviation is 10 words. **c** Temporal distribution of the number of videos (gray) and comments (black) in our database.

We acquired 938,854 comments. Using the open-source DLATK Python codebase^26^, we filtered out duplicated comments, non-English comments, and short comments (5 words or fewer). The cleaning process yielded our final dataset that contains 569,581 comments. The average number (SD) of comments per video is about 15 comments (72), and the average length of a comment is 20 words (10) (Fig. 1a, b). The comments were posted between January 2021 to June 2025 (Fig. 1c). The difference in end dates for videos (December 2024) and comments (June 2025) is because after being posted, videos can continue circulating and accumulating comments (Fig. 1c). Though we did not explicitly query for comments that contain our opioid keywords, the comment corpus contains these keywords (Supplementary Fig. 1).

### Correlation analysis of TikTok topics and CDC opioid overdose mortality

#### Overall population correlations

**Fig. 2:**
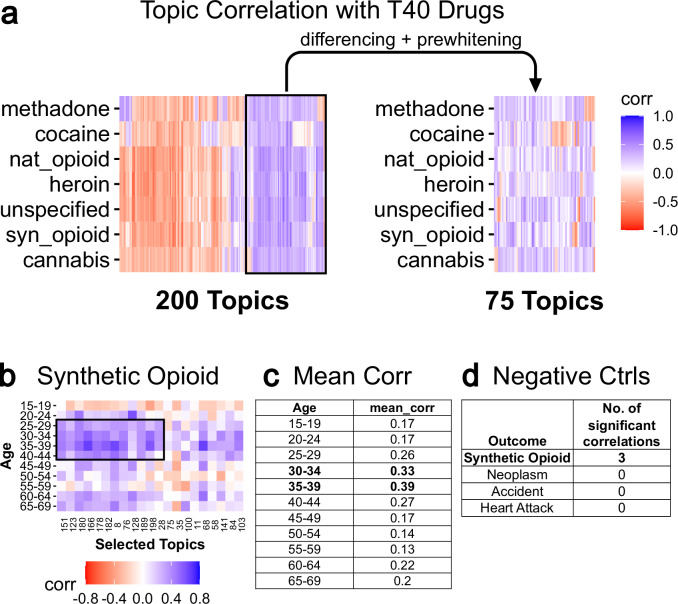
Cross-correlation analysis of TikTok topics with CDC opioid overdose mortality rates. **a** Cross-correlation of all 200 TikTok topics (found in Supplementary Table 1) and deaths caused by substances under the T40 ICD-10 code (LEFT). The *y*-axis represents drug categories under the T40 ICD-10 code: methadone (T40.3), cocaine (T40.5), nat_opioid for natural and semi-synthetic opioids (T40.2), heroin (T40.1), syn_opioid for synthetic opioids (T40.2), and cannabis (T40.7). The *x*-axis represents TikTok topics (topic names are not listed). For the topic cluster that was positively correlated (75 topics listed in Supplementary Note 4), we also computed their correlations after differencing (to ensure stationarity) and prewhitening (to eliminate autocorrelations) (RIGHT). Twenty-six of these topics still maintained a positive correlation after detrending and prewhitening. **b** TikTok topic correlations with opioid overdose deaths categorized by age for synthetic opioids. TikTok topics are most correlated with deaths among people between 30 and 39 years old. Wordclouds for topics are shown in Supplementary Table 1. These correlations are not controlled for multiple comparisons. **c** Mean of absolute correlation between TikTok topics and synthetic opioid overdose death rate (by age group). Ages between 30 and 39 had the highest correlation. **d** Number of significant age-stratified correlations between TikTok topics (**b**) and different CDC death outcomes. We only observe significant TikTok correlations with synthetic opioid overdose deaths. The topics with significant correlations are topics 8, 68, and 166 (see Supplementary Table 1 for word clouds).

We modeled 200 topics from the TikTok comments (using LDA) and aggregated topic scores by month. Correlating the time series of all 200 topics with drug subcategories under the T40 ICD-10 code (for months between January 2022 and June 2024), we observed that some TikTok topic scores are positively correlated with these CDC mortality rates, while others are negatively correlated or did not correlate with these drug outcomes (Fig. 2a; see Supplementary Table 1 for topic wordclouds). Positive correlation coefficients ranged between +0.0024 and +0.8209. To avoid spurious correlations, we detrended and prewhitened the time series signals. After detrending and prewhitening both time series data and correlating them, some TikTok topics still maintained correlation values above 0 (Fig. 2a), suggesting that positive correlation remained after preprocessing. Positive preprocessed correlations ranged between +0.0009 and +0.6611.

#### Age-specific correlations

Several topics are most correlated with synthetic opioid overdose mortality rates for ages between 25 and 44 years (Fig. 2b). Computing the mean of absolute correlations across topics for each age group, we observed that 30-39 years had the highest correlation (*r* > 0.3) (Fig. 2c).

Using a two-tailed *z*-test, we did not observe any statistically significant age-stratified correlations between the TikTok topics and negative control outcomes (i.e., neoplasm, accident, and heart attack) (Fig. 2d).

### Predictive modeling of synthetic opioid overdose mortality

We built ARIMA models to forecast synthetic opioid overdose death rates per 100,000 people over six-month horizons. When fitted solely to CDC mortality data, the ARIMA model achieved a mean absolute error (MAE) of 0.38 (deaths per 100,000 people) for 6-month forecasting horizons across 10 months between September 2023 and June 2024.

**Fig. 3:**
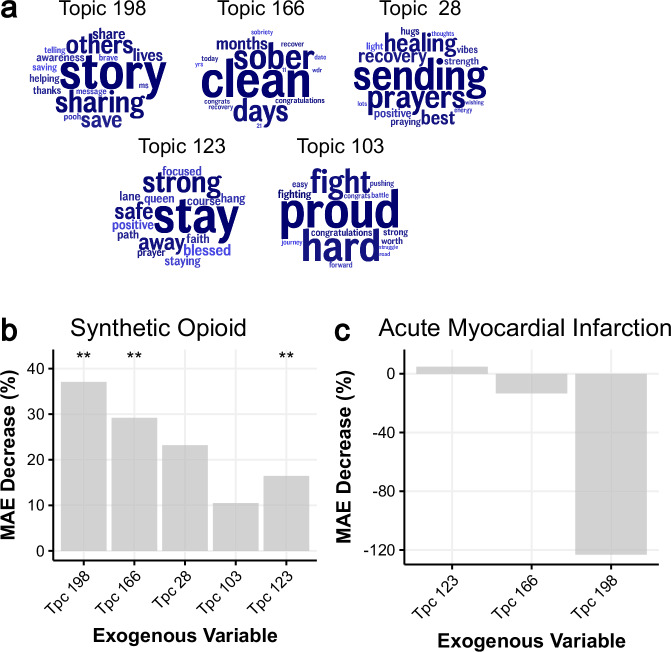
ARIMA forecasts of opioid overdose mortality rates incorporating TikTok topics. **a** Wordclouds of topics incorporated into ARIMA models. Topics 198, 166, and 123 led to a statistically significant improvement in forecasting the synthetic opioid overdose death rate. **b** Percentage decrease in MAE for ARIMA models that forecast synthetic opioid overdose death rates. Incorporating topics decreased MAE (relative to the error of the ARIMA model fitted solely on CDC data); however, only topics 198, 166, and 123 caused a statistically significant decrease (*p* < 0.01). **c** Percentage decrease in MAE for ARIMA models that forecast acute myocardial infarction (heart attack) death rates. Incorporating topics 198, 166, and 123 into the models either increased MAE or did not cause a significant decrease in MAE.

Topics 198, 166, 28, 103, and 123 (Fig. 3a) are the most correlated with the synthetic opioid overdose death rate, and they lead the CDC synthetic opioid overdose mortality rate by 3 months (i.e., opioid overdose death in month *t* is correlated with topic score in month *t-3*). We applied a 3-month lag to each of them and incorporated them into separate ARIMA models as exogenous variables. The models achieved MAEs of 0.24, 0.27, 0.29, 0.34, and 0.32 (deaths per 100,000 people) for topics 198, 166, 28, 103, and 123, respectively. These correspond to 37%, 29%, 24%, 11%, and 15% reduction in MAE (Fig. 3b). Only topics 198, 166, and 123 yielded statistically significant improvements (Wilcoxon Signed Rank test, *p* < 0.01) (Fig. 3b and Supplementary Fig. 2). Interestingly, all three topics are thematically related to recovery from SUD (Fig. 3a). We also examined the most negatively correlated topics and did not observe a significant improvement.

As negative control exposures, we used three topics (topics 127, 149, 187) that are uncorrelated with synthetic opioid overdose death rate as exogenous variables (Supplementary Fig. 3d). Including these topics made the model performance worse (MAE > 0.38) (Supplementary Fig. 3b). We also incorporated different lagged versions of Topic 198 (the topic with the best MAE) into ARIMA models, using lags ranging from one to six months (Supplementary Fig. 3a). The 3-month lagged model achieved the lowest MAE (Supplementary Fig. 3a), reinforcing that this TikTok topic leads CDC mortality rate reports by approximately three months.

To test how specific the best-performing topics (topics 198, 166, 123) are to opioid overdose death rates, we incorporated them into ARIMA models to predict heart attack (acute myocardial infarction) death rates among people over 60. Compared to the CDC-only heart attack model, the CDC + topic heart attack models performed comparable (no statistically significant decrease, Wilcoxon Signed Rank test *p* > 0.1) or worse (higher MAE) (Fig. 3c).

### Themes of opioid comments on TikTok

We filtered the 200 LDA-generated topics based on their relevance to SUD. The filtered topics (*n* = 47 topics) spanned a broad range of themes from drug seeking to drug withdrawal. We grouped them into four major categories: Use, Source, Recovery, Harm Reduction, and Loss. There were 47 topics across all four non-overlapping categories, with 23% being Use topics, 11% being Source topics, 43% being Recovery topics, 9% being Harm Reduction topics, and 15% being Loss topics.

**Fig. 4:**
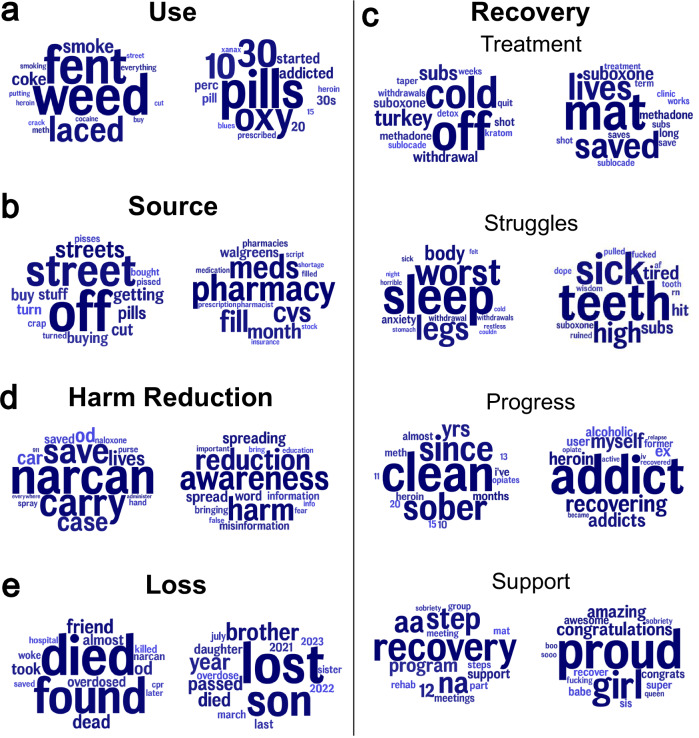
Categories of topics generated from LDA modeling. **a** Representative topics in the use category. This category contains topics that pertain to opioid use. Representative topics are topics 87 and 188. **b** Representative topics in the Source category. These topics pertain to how opioids are acquired. Topics shown are topics 1 and 133. **c** Representative topics in the recovery category. This category includes the following subcategories: treatment (topics 105 and 135), recovery duration or progress (topics 68 and 11), recovery struggles (topics 83 and 37), and support (topics 89 and 182). **d** Representative topics in the harm reduction category. This category contains topics that pertain to minimizing the negative effects of opioid use. Topics shown are topics 15 and 128. **e** Representative topics in the loss category. The loss category contains topics that pertain to loss of lives as a result of the opioid epidemic or opioid overdose. The topics shown are topics 177 and 107.

The Use category contains topics that denote the use of opioids and drugs more generally. This encompasses both prescribed use and non-medical use. Topics under this subcategory usually contain drug names (e.g., “fentanyl,” “oxy,” “perc,” “cocaine”), active verbs that denote use (e.g., “smoke,” “take,” “prescribe,” “hooked,” “addicted”), apparatus for opioid misuse (e.g., “foil,” “burn,” “pill,” “patches,” “skin”), and drug dose amounts and units (e.g., “10,” “30,” “mg”) (Fig. 4a and Supplementary Fig. 4). An example comment associated with this category is “*Imma eat both fenty xans low-key litty*” (which means “*I am going to take both fentanyl and xanax, kind of great*”). List of topics includes 38, 39, 67, 78, 87, 96, 120, 163, 165, 188, and 199 (Supplementary Table 2).

The Source category contains topics that portray how opioids are acquired. This category also contains both licit and illicit use. In this category, we see two sources: medical establishments and the streets. Some words that allude to sourcing opioids from medical establishments include “pharmacy,” “prescription,” “fill,” and “doctor”. Words that denote acquiring opioids from the streets include “street,” “buy,” “off,” “stuff,” “dealer” (Fig. 4b and Supplementary Fig. 4). An example comment is “*meantime people are turning to the streets out of desperation and getting poisioned”*. List of topics includes 1, 51, 133, 139, and 196 (Supplementary Table 2).

The Recovery category denotes withdrawal from or treatment for opioid use disorder (OUD). We subcategorized the Recovery category into the following: *Treatment, Struggles, Progress*, and *Support* (Fig. 4c and Supplementary Fig. 5).

- Treatment: contains topics regarding active withdrawal from and treatment for OUD or SUD. In this subcategory, we observed two approaches to OUD recovery: abrupt stop in opioid usage and using medication-assisted treatment (MAT). The co-occurrence of words such as “cold,” “turkey,” “quit,” “detox,” and “withdrawal” suggests comments related to the former type of OUD treatment. For MAT, we see the co-occurrence of words related to treatment and therapeutics, such as suboxone (also referred to as “subs”) and methadone. An example comment from this category is “*I'm going through withdrawals cold turkey from 8mgs subbetex day two*,”. List of topics includes 19, 86, 90, 105, and 135.
- Struggles: contain topics that allude to the struggles of recovering from SUD. The topics contain words like “sober,” “fight,” “struggle,” and symptoms like “anxiety”. Some of the topics also reference body parts (e.g., “leg,” “stomach,” “teeth”). An example comment associated with this subcategory is: “*Restless legs and hot/cold flashes and sweating were the worse for me. And runny nose and eyes. Sneezing. Stomach cramps*”. The list of topics includes 37, 83, 93, 155, and 186.
- Progress: contains topics about recovery progress and how long an individual has been sober from opioids or other substances. The topics typically contain words such as “clean,” “sober,” and longer units of time (e.g., day, month, year). An example comment is “*been clean 7 months on 1-25-24, my clean date is 6-25-23*”. List of topics includes 11, 30, 68, 94, 141, and 166.
- Support: contains topics about supporting and encouraging other individuals’ recovery journeys from SUD. Dominant words for this category include: “proud”, “fight”, “sobriety,” “congratulations,” etc. Example comment is “*congratulations stay clean itsnwell worth it*”. The list of topics includes 89, 154, and 178.

The Harm Reduction category contains topics about harm reduction (Fig. 4d). Frequent words in this category include “narcan,” “awareness,” “education,” etc. Example comment is: “*Narcan. Everyone needs to carry narcan*”. List of topics includes 15, 123, 138, and 170 (Supplementary Table 2).

Lastly, the Loss category contains topics that portray that people are dying from opioid overdose (Fig. 4e and Supplementary Fig. 6). Words that dominate this subcategory are “lives,” “lost,” “epidemic,” “overdose,” etc. An example comment is “*Fentanyl is killing a generation of our kids*”. List of topics includes 24, 25, 54, 57, 107, 125, 177.

### POV analysis of opioid comments on TikTok

**Fig. 5:**
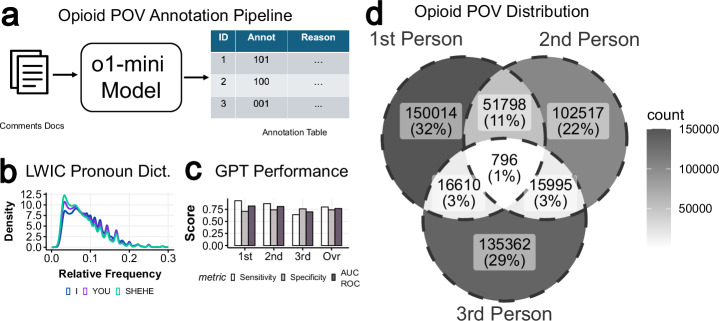
POV of opioid comments on TikTok. **a** Workflow used to annotate the primary POV with respect to opioid use in comments. We query the o1-mini reasoning model with a prompt instruction alongside the comments to be annotated. This annotation pipeline returns three objects per comment: ID, GPT Annotation, and GPT Reason. The ID is a unique identifier for each comment; GPT Annotation is the POV annotation GPT assigns to the comment, and GPT Reason is the reasoning behind GPT’s annotation. **b** Density distribution of non-zero LIWC pronoun dictionary scores. The density distributions of the I (1st person pronoun perspective), YOU (2nd person pronoun perspective), and SHEHE (3rd person pronoun perspective) dictionaries are comparable, suggesting that there is a fair representation of all three pronoun dictionaries in our TikTok comment dataset. **c** GPT o1-mini’s performance in annotating the opioid use POV of 500 examples manually annotated by authors. The model’s sensitivity, specificity, and AUC-ROC scores are above random (0.5) for each POV (i.e., 1st, 2nd, and 3rd person opioid perspectives). The overall performance across all three POVs is also above 0.5 (micro-averaged). **d** Distribution of opioid perspectives from GPT’s annotations. The majority of comments carry only one opioid POV. The proportions of 1st and 3rd person opioid POV are close (32% and 29%), followed by the proportion of 3rd person POV (21%).

To further characterize the nature of comments on opioid-related videos on TikTok, we analyzed the POV with which comments were written (Fig. 5). Knowing the distribution of POV of opioid comments on TikTok (e.g., proportions of personal accounts with opioid versus proportions of dialogs about opioids versus proportions of accounts of opioid use in the community and beyond) can inform what kind of insights can be gleaned from TikTok.

We analyzed opioid POV using the LWIC2015 pronoun dictionary (I vs YOU vs SHEHE) and the GPT o1-mini language model (Fig. 5a). There are almost equal distributions of the three pronoun dictionaries (Fig. 5b). We also evaluated GPT’s performance in annotating the primary POV (Fig. 5c) and subsequently used it to annotate the comments (see POV Annotation in Methods for more details). In terms of primary POV with regards to opioid use, about 83% of the annotated opioid related comments are 1st, 2nd, or 3rd person only (Fig. 5d). There are roughly equal proportions of 1st and 3rd person opioid POV comments (32% and 29%, respectively), and a slightly lower proportion of 2nd person opioid POV comments (22%) (Fig. 5d). For comments that contained two opioid POVs, there were about 3.5 times more comments containing 1st and 2nd person opioid POV (11%) than there were comments containing 1st and 3rd person opioid POV (3%) or 2nd and 3rd person opioid POV (3%) (Fig. 5d).

## Discussion

Our findings support three major conclusions. First, opioid topics modeled from TikTok comments correlate with and are predictive of real-world opioid overdose deaths (Figs. 2 and 3). We also showed age specificity in these correlations. Second, opioid comments on TikTok comprise at least five broad themes: Use, Source, Recovery, Harm Reduction, and Loss (Fig. 4 and Supplementary Figs. 4–6). Third, TikTok hosts varied accounts of opioid use, including personal accounts, as well as second and third person narrations (Fig. 5), suggesting that TikTok can be a useful data source for researchers studying opioid narrations from different perspectives.

The TikTok topics reflect known phenomena about opioid misuse and recovery from OUD. Under the Use category, we see topics that contain words (such as foil and patches) that hint at comments related to opioid misuse via inhalation (known as “chasing the dragon”) or transdermal patches (Supplementary Fig. 4). In the same category, there are topics in which opioids co-occur with other drug names (Fig. 4a and Supplementary Fig. 4), suggestive of comments around opioid use with other drugs. The polysubstance use of opioids and other drugs has become popular and is referred to as the fourth wave of the opioid overdose crisis^27^.

The Struggles subcategory contains topics that allude to challenges associated with OUD recovery (Fig. 4b and Supplementary Fig. 5). In addition to containing recovery-related keywords (such as withdrawal), these topics contain psychological words (such as anxiety), which are suggestive of mental health challenges, and body parts (such as legs, teeth, etc), which are suggestive of physical health challenges. These topic keywords are consistent with withdrawal symptoms reported to be associated with OUD recovery^28^.

Under the Progress subcategory, some topics contain words about being sober and units of time (e.g., weeks, months, years, etc) (Fig. 4b and Supplementary Fig. 5). This is because TikTok users undergoing sobriety use TikTok as an avenue to share information about their SUD recovery progress; this information includes sobriety date and duration of sobriety. This practice is common in many SUD recovery groups, such as alcoholics anonymous (AA) and Narcotics Anonymous (NA)^29^.

Humphreys et al. and many others have shown that the support of recovery groups (such as AA) and personal networks (such as friends and family) improves the outcome of recovery^30–32^. We see this kind of support in TikTok comments. The topics under the Support subcategory contain words (such as proud, congratulations, and strong) that denote comments aimed to cheer other people through their recovery process (Fig. 4b and Supplementary Fig. 5).

We found that some of the TikTok topics (topics listed in Supplementary Note 4) are positively correlated with CDC overdose death reports (Fig. 2a). This could be because the occurrence of opioid overdose events may influence opioid conversations (e.g., news reporting, people sharing personal experiences) on TikTok. Though we only acquired opioid-related comments data for this study, we observed that some topics generated from the comments corpus also positively correlated with non-opioid drugs (such as cocaine) mortality (Fig. 2a). Polysubstance use is what is likely driving the positive correlation between opioid chatter on TikTok and non-opioid deaths^33,34^.

As of May 2025, 55% of TikTok users based in the US were between the ages of 25 and 44 years^20^. As such, TikTok topics were most correlated with synthetic opioid overdose mortality among a subset of this age range (i.e., between 30–39 years) (Fig. 2b, c), though opioid deaths were distributed across all age groups in our dataset (Supplementary Fig. 7).

Accurately forecasting opioid mortality rate is essential to monitoring the opioid epidemic and effectively deploying resources to mitigate the crisis^7,35^. We showed that TikTok comments can help improve opioid overdose mortality rate forecasting (Fig. 3). Compared to fitting ARIMA on only CDC synthetic opioid overdose death rate, fitting ARIMA on CDC data and topics 198, 166, and 123 (recovery topics modeled from TikTok comments) improved 6-months overdose death rate forecast MAE by 37%, 29%, and 16%, respectively (with statistical significance, Wilcoxon Signed Rank test *p* < 0.01) (Fig. 3a, b and Supplementary Fig. 2). This suggests that, though the official CDC opioid overdose death report delays by 6 months, we can obtain more accurate near real time estimates of opioid overdose deaths rates by incorporating TikTok topics. We systematically ablated our forecasting setup and found that all modifications decreased performance (Fig. 3c and Supplementary Fig. 3), confirming that the components of our prediction setup are specific to forecasting synthetic opioid overdose death rates.

These findings underscore how useful TikTok could be in tracking the opioid crisis. While the majority of social media-based analysis of opioids has been conducted on Twitter and Reddit^8,10,14,36^, this work demonstrates TikTok’s utility for opioid epidemic monitoring even without video and audio processing. To our knowledge, this study is the first qualitative study of opioid-related comments on TikTok and also the first quantitative study showing that TikTok comments can predict opioid overdose deaths. Since TikTok is popular among young adults^20^, it provides the opportunity to capture younger demographics in opioid surveillance.

Inspecting the topics (198, 166, and 123) that showed the most statistically significant improvements in ARIMA forecasts, we found that all three topics were related to SUD recovery (Fig. 3a). They contain words such as “clean”, “sober”, “save”, “awareness”, “stay”, “strong” and were positively correlated with overdose mortality rates. This is an interesting finding, as one might expect topics about opioid misuse to be the topics that are most positively correlated with overdose mortality rates and improve forecasting the most. We hypothesize that this could be because people tend to get into recovery when their SUD has gotten very severe^37^. At this point of SUD, overdose is more likely^38^. This phenomenon may also be explained by the deaths of friends, family, or broader community members from opioid overdose, motivating recovery efforts in those surviving.

After establishing that TikTok comments contain relevant opioid themes via topic modeling and ARIMA forecasting, we evaluated the primary POV with which these comments were authored. From the LIWC 2015 pronoun dictionary extraction, we observed that there is almost equal prevalence of first person (I), second person (YOU), and third person (SHEHE) pronoun perspectives (Fig. 5b and Supplementary Note 5). We also observed the presence of 1st, 2nd, and 3rd person opioid POVs (Fig. 5d). This means TikTok users not only use the platform for sharing opioid news (3rd person opioid POV) but also share their personal opioid experiences (1st person opioid POV). The significant proportion of 2nd person opioid POV also suggests that users engage with each other’s opioid experiences. These engagements can be in the form of advice, support, counseling, etc^39,40^. This fair representation of all three pronoun and opioid perspectives makes TikTok an appealing monitoring tool for several types of researchers, including researchers interested in personal accounts of opioid experiences, researchers interested in how users interact with the opioid experience of other users, and researchers interested in how opioid-related news is discussed and spread. Also, accounting for the 2nd and 3rd person perspectives can expand data samples over accounting for just the 1st person perspective.

Recent restrictions on the use of the PushShift API (a third-party Reddit scraper API)^41^ and the Twitter API limit the long-term utility of Reddit and Twitter for opioid pharmacovigilance^8,21^. In contrast, TikTok has a free API available to researchers, making the platform an appealing alternative for opioid tracking. As of the time of writing, TikTok faces a potential ban in the US if it fails to comply with federal ownership requirements. While numerous government officials and segments of the public oppose a TikTok ban, the ultimate resolution of this policy debate remains to be determined. There has also been a growing amount of internet traffic directed towards other short-form video platforms such as Instagram (reels) and YouTube (shorts)^42^. Therefore, the approach employed in this study (querying and analyzing comments under video posts) may be generalizable to these platforms, as the comment section can contain rich discourse and conversation.

A limitation of this study is that we assume that comments have the same geotags as video posts (i.e., the US). Since TikTok is still a new tool to monitor perceptions about opioid use and the opioid crisis, there is a dearth of studies on how to geolocate TikTok comments and videos beyond the country level (to state, county, or city level). Being able to geolocate comments will facilitate more granular analysis to identify potential hotspots within the US that will benefit the most from interventions. Therefore, this presents an avenue for future work to investigate strategies to geotag TikTok data at finer levels of resolution.

We also did not explicitly filter out bot-generated comments, as distinguishing between a human-authored text and a bot-authored text remains an open problem^43^. Prior studies estimate that 9%–20% of posts on platforms such as Twitter are generated by bots^43^, thus we acknowledge that our dataset likely contains bot-generated content. Nevertheless, incorporating these comments still yielded a statistically significant improvement in ARIMA forecasts (Fig. 3b).

Finally, in our ARIMA ablation tests, we used outcomes such as neoplasm, accident, and heart attack as negative control outcomes. Although these outcomes are not expected to be directly related to opioid use/misuse, we do acknowledge the possibility of indirect associations. Despite this, TikTok opioid topics did not improve the forecast for these outcomes (Figs. 2d and 3c).

While leveraging social media data offers valuable insights, it is crucial to address ethical considerations. As we monitor social media chatter to track the opioid crisis, it is important to protect the privacy and anonymity of social media users, especially given the sensitive nature of SUDs^44,45^. Our aim in this work was to identify correlations with and forecast nationwide overdose death rates, not to identify individuals at risk of overdose. We do not have access to user details of comment authors and do not report any personal identifiable information. We perform all analyses using aggregate data. This ensures that our research contributes to understanding the opioid epidemic while maintaining strict ethical standards and respecting user privacy.

## Methods

### Data acquisition

We acquired TikTok comment data through the TikTok Research API. We first queried the API for videos that met the following criteria: (i) the video description matches a word within a list of opioid keywords (Supplementary Note 1), (ii) the video author’s country is the US. We limited our search to the US because we performed downstream validation analyses using US overdose death statistics.

Our opioid keyword list (Supplementary Note 1) was adapted from Carpenter et al.^21^ and contains formal and informal opioid keywords. Formal opioid keywords are official terms used to refer to opioids (e.g., “fentanyl”). Informal opioid keywords are slang terms, abbreviations, or misspellings commonly used on social media or in everyday language to refer to opioids (e.g., “fent”). Out of a list of 28 keywords, we got matches for 24 keywords within the description field (Supplementary Fig. 1a) and acquired 48,306 video IDs of videos posted between January 2021 and December 2024.

We used the TikTok Research API to download all comments associated with these video IDs to create a TikTok comments dataset.

### Topic modeling and LDA

We used Latent Dirichlet Allocation (LDA)^46^ to discover themes within the comments dataset. LDA is an unsupervised topic modeling algorithm that represents each document as a mixture of latent topics, with each topic defined by a distribution over words. It assumes that each document’s topic mixture is drawn from a Dirichlet prior with parameter α. For each word position in the document, a topic is first sampled from this mixture, and then a word is sampled from the chosen topic’s word distribution (which is also drawn from a Dirichlet prior with parameter β)^47^. This hierarchical Bayesian formulation allows LDA to capture both document-level thematic heterogeneity and the polysemy of words across different contexts^46,47^. The marginal probability of observing a word $w$ in a document $d$ is given as:

1 $$p(w\,|\,d)=\mathop{\sum }\limits_{k=1}^{K}p(w\,|\,z=k)\,p(z=k\,|\,d)$$

where $z$ denotes the latent topic assignment, $k\epsilon \{1,\,...\,,{K}\}$ indexes the $K$ (predefined number of topics), $p({w\; |\; z}=k)$ is the word distribution for topic k, and $p(z={k\; |\; d})$ is the topic proportion for document $d$. We ran LDA using the MALLET package^48^ as implemented by the DLATK codebase^26^.

We modeled 200 topics; we found that modeling 200 topics yielded coherent and appropriately granular themes. After topic modeling, we extracted the relative frequency with which each comment expressed the 200 topics. This yielded 200 values for each comment, summarizing the comment as a distribution over 200 topics.

### Topic grouping

IAS (a Bioengineering PhD candidate with previous relevant research experience^21^) reviewed each of the 200 topics to determine which topics were relevant to SUD (Supplementary Table 1). We classified a topic as relevant to SUD if it included keywords or thematic content indicative of drug misuse or key concepts associated with substance use (Supplementary Table 1). These concepts included drug acquisition, withdrawal effects, etc.

This led to a shortlist of 47 topics. Two authors, I.A.S. and A.L. (an expert on addiction medicine and the opioid epidemic), reviewed these topics and identified five broad categories that encompass the majority of them: use, source, recovery, and loss (Supplementary Table 2). Each topic was manually examined along with representative comments (those with high relative frequencies for the topic) to determine the most appropriate category. To ensure thematic consistency, we further organized some categories into subcategories.

### Monthly TikTok topic score normalization

We aggregated the comments by month (on average, relying on about 15,068 comments per month). We computed the relative frequency with which each monthly aggregate of comments expresses each of the 200 topics. During this calculation, topic scores are normalized by the total number of words (one-gram tokens) in a month to yield topic scores per 100,000 words.

### CDC overdose deaths data and normalization

We acquired US monthly overdose data from the CDC WONDER platform^49^. CDC WONDER is an online system that the Centers for Disease Control and Prevention (CDC) uses to disseminate epidemiological data and statistics. In our query, we specified the Underlying Cause of Death (UCD) to be Drug-induced Causes (ICD-10 codes: X40-X44, X60-X64, X85, Y10–14) and the Multiple Causes of Death to be the T40 ICD-10 code, which is a subset of overdose deaths caused by poisoning from narcotics and psychodysleptics. The T40 ICD-10 code is subcategorized into deaths caused by the following substances: heroin (T40.1), methadone (T40.3), natural and semi-synthetic opioids (T40.2), synthetic opioids (T40.4), cocaine (T40.5), and cannabis (T40.7). In the demographic section, we specified that deaths be categorized by 5-year age groups. We normalized the number of deaths by monthly US population estimates from the US Census Bureau^50^. This yielded the age-specific crude rate of overdose deaths per 100,000 people. Crude death rate for an age group is the total number of deaths for that age group divided by the total population.

As negative control outcomes^51^, we acquired the US monthly deaths caused by neoplasm or cancer (C00–C997), accidents (V40–V49), and heart attack (I21). We chose these outcomes as negatives because TikTok opioid comments should not be related to these outcomes. We also normalized these values with census data to yield age-specific crude death rates per 100,000 people.

### CDC mortality rate and normalized TikTok topic score correlation

For each TikTok topic, we conducted cross-correlation analyses comparing the TikTok topic time series with CDC mortality rates for all ICD-10 T40 subcodes deaths. T40 subcodes are deaths due to poisoning by heroin, methadone, natural and semi-synthetic opioids, synthetic opioids, cocaine, and cannabis. To account for potential lag, we examined temporal relationships by allowing CDC mortality timeseries data to lead TikTok topic data by 0 to 6 months. That is, for each pair of topic and T40 subcode (drug class), we calculated Pearson correlations between TikTok topic rates at month *t* and corresponding CDC drug death rate at months *t*, *t* + 1, *t* + 2, *t* + 3, *t* + 4, *t* + 5, and *t* + 6, yielding seven correlation coefficients per comparison. We report the maximum correlation coefficient and its associated lead time (in months). To mitigate spurious correlations due to underlying time series trends, we applied differencing to both time series data (to ensure the signals are stationary)^8^, applied prewhitening (to eliminate autocorrelations)^52^, and then performed another set of correlations. Some CDC outcomes required first-order differencing to be stationary, while others required second-order differencing. We tested for stationarity using the Augmented Dickey–Fuller (ADF) test.

We excluded months before January 2022 from the correlation analysis because those months had lower numbers of comments (Fig. 1c). This could be because we sampled fewer videos from 2021 (Fig. 1c). Because CDC’s 2024 data is still provisional at the time of writing, we only included the first 6 months of 2024 in our analysis. Thus, our cross-correlation analysis between normalized TikTok topic scores and CDC overdose mortality rate spanned January 2022 to June 2024.

### CDC mortality rate by age and TikTok topic score rate correlation

From the previous cross-correlation analysis, we obtained 200 topic correlations per drug category (e.g., T40 subcode). These are correlations between TikTok topic time series and non-stratified drug mortality rate time series. For each drug category, we filtered for topics with correlation coefficients over 0.4 to create a shortlist of positively correlated topics. We then examined correlations between this filtered topic set and mortality rates stratified by age group per drug category.

For each topic and drug category pair, we computed both trended and preprocessed (i.e., after detrending and prewhitening) correlations across age-stratified groups. Since the topics in this age correlation analysis were selected based on their highest correlations with the overall drug overdose death rate trend, we report their processed correlations to isolate the relationship independent of temporal trends.

We also computed age-stratified correlations between the filtered topic set and negative outcomes: neoplasm or cancer (C00–C997), accidents (V40–V49), and heart attack (I21).

To evaluate the significance of the computed correlation coefficient across age groups and topics for each outcome, we computed a *p*-value for each coefficient using a two-tailed *z*-test^52^. We assumed that the correlation coefficients follow a normal distribution with standard error of 1/sqrt(*n*), where n represents the number of datapoints (here, months)^52^. Each of these *p*-values was compared to a Benjamini–Hochberg adjusted threshold.

### Autoregressive integrated moving average (ARIMA) modeling

Using a moving origin forecast approach and the Python pmdarima auto_arima function, we fit an ARIMA model on the synthetic opioid overdose death rates. We only focus on synthetic opioids because they are responsible for the majority of opioid overdose deaths. Given the synthetic opioid overdose mortality rate for 12 months, we used the ARIMA model to autoregressively predict mortality rates for the next 6 months. We computed the absolute error between the ARIMA model’s prediction for the sixth month and the CDC’s reported synthetic opioid death for that month. We performed this six-month forecast to simulate the 6 months delay in CDC official overdose death reporting^8,35^. This showed how well current CDC overdose deaths alone predict future overdose deaths. With this setup, we were able to generate and evaluate predictions for 10 months (September 2023 to June 2024), relying on data from January 2022 to June 2024.

We introduced TikTok topic distributions as exogenous variables to the ARIMA model to evaluate their impact on prediction performance. We tested the five topics that were most correlated with the synthetic opioid death rate and whose time series distributions led the CDC’s synthetic opioid mortality time series by at least one month. These criteria ensured that we were selecting topics correlated with future CDC deaths, which is helpful for forecasting. The time series of all five shortlisted topics leads the CDC synthetic opioid death rate time series by 3 months. We lagged each topic by 3 months and built an ARIMA model on the CDC synthetic opioid overdose data and the 3 months lagged topic distribution as an exogenous variable. With each ARIMA model, we generated overdose death rate forecasts over 6-month horizons and evaluated the absolute errors of these forecasts in the sixth month.

We tested the significance of the impact of introducing TikTok signals on prediction error using the Wilcoxon Signed Rank test with a Bonferroni-adjusted threshold.

### Linguistic inquiry and word count (LIWC) pronoun dictionaries extraction

LIWC^53^ is a text analysis tool that quantifies the frequency of predefined linguistic dictionaries within text. It has been widely applied to analyze patterns of language use in large text corpora^36,54^. We extracted the relative frequency of three pronouns dictionaries (“I,” “YOU,” and “SHEHE”) provided by LIWC 2015^53^. We used these relative frequency values to represent interpersonal frames of comments. The “I” dictionary represents the first person pronoun perspective, “YOU” represents the second person pronoun perspective, and “SHEHE” (a combination of “SHE” and “HE”) represents the third person pronoun perspective.

### GPT opioid POV annotation

Beyond counting the frequencies of pronouns, we used the GPT o1-mini model^55^ to annotate the perspective of comment posts in relation to who is implied to be using opioids. We will refer to this as the opioid POV. Consider this example: “*I think you took poisoned fentanyl*”. Although written from a first-person perspective, its mention of opioids is in the second-person frame, as it references another individual’s opioid experience. Consider another example: “*You are convinced they respond better to morphine*”. Although the comment adopts a second-person narrative style, it refers to a third person’s opioid experience and is thus classified as a third-person perspective with respect to opioids (or third-person opioid POV). It is worth noting that comments can have multiple opioid POV. For example, the comment “*I had a fun experience with perc, sorry you did not enjoy it*” refers to both a first-person and a second-person opioid POV. More examples are shown in Supplementary Tables 3 and 4.

We iteratively created a prompt (see Supplementary Note 2) to make the GPT o1-mini model (accessed via the OpenAI Chat Completions API from Microsoft Azure) perform the opioid POV annotation using 28 comments (Supplementary Table 4). We evaluated the performance of the combination of model and prompt instruction on 500 comments manually annotated by two authors. The annotations had a Cohen’s Kappa between 0.67 and 0.75, indicating substantial agreement between annotators according to Landis and Koch’s^56^ widely used interpretation (see Supplementary Note 3 for details on manual labeling and inter-rater agreement). These comments were randomly selected and held out from prompt development.

## Supplementary information

Supplementary Information

## Data Availability

The data used and generated from this study are publicly available. Details on accessing this data can be found here: https://github.com/Small-Samori/tiktok-opioid-tracking.git.

## Code availability

The code for this study is publicly available at this link: https://github.com/Small-Samori/tiktok-opioid-tracking.git.
