# Supplementary material for: TikTok is a valuable data source for tracking the opioid crisis
Source: NPJ Digit Med. 2026 Apr 27;9:501. doi: 10.1038/s41746-026-02654-x (PMC13324712; doi:10.1038/s41746-026-02654-x)
Supplement: Supplementary file 1 — Supplementary Information [file 41746_2026_2654_MOESM1_ESM.pdf]

SUPPLEMENTARY FIGURES

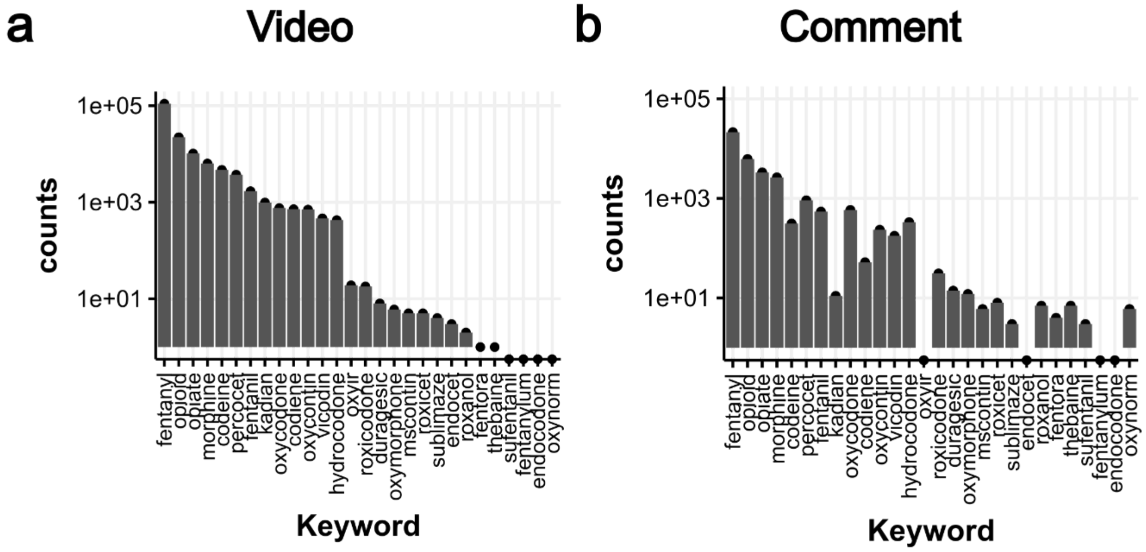

Fig. S1: Keyword Counts

Occurrences of query keywords in **a**: video descriptions, **b**: comment data.

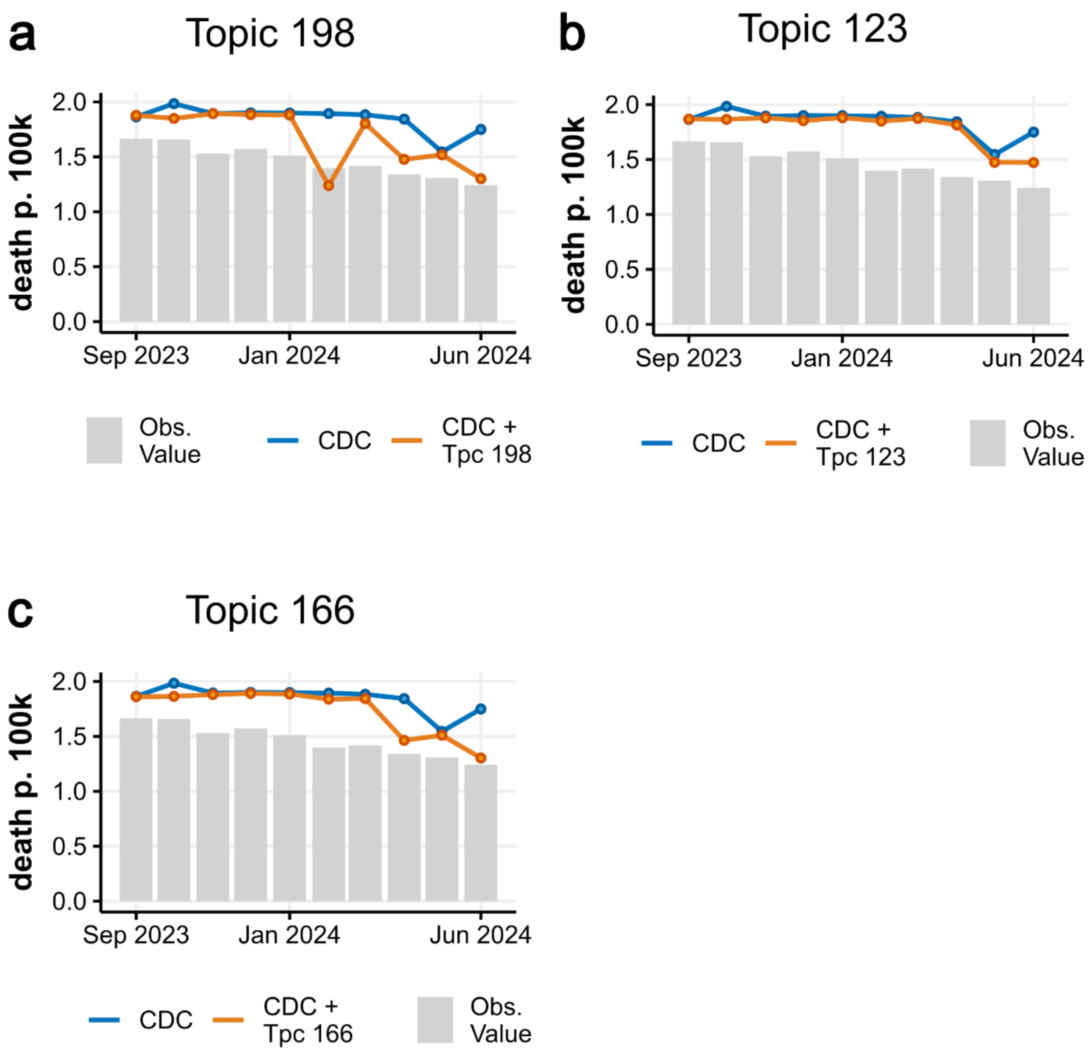

Fig. S2: Comparison of Predicted and Observed Synthetic Opioid Mortality Rate
Comparison of reported synthetic opioid overdose deaths (gray barcharts) and ARIMA forecasts (colored trendlines). Blue trendlines represent forecasts for the ARIMA model fitted on only CDC data, while orange trendlines represent forecasts for the ARIMA model fitted on CDC data with TikTok topics as exogenous variables. Topics 198 (**a**), 123 (**b**), and 166 (**c**) were used as exogenous variables.

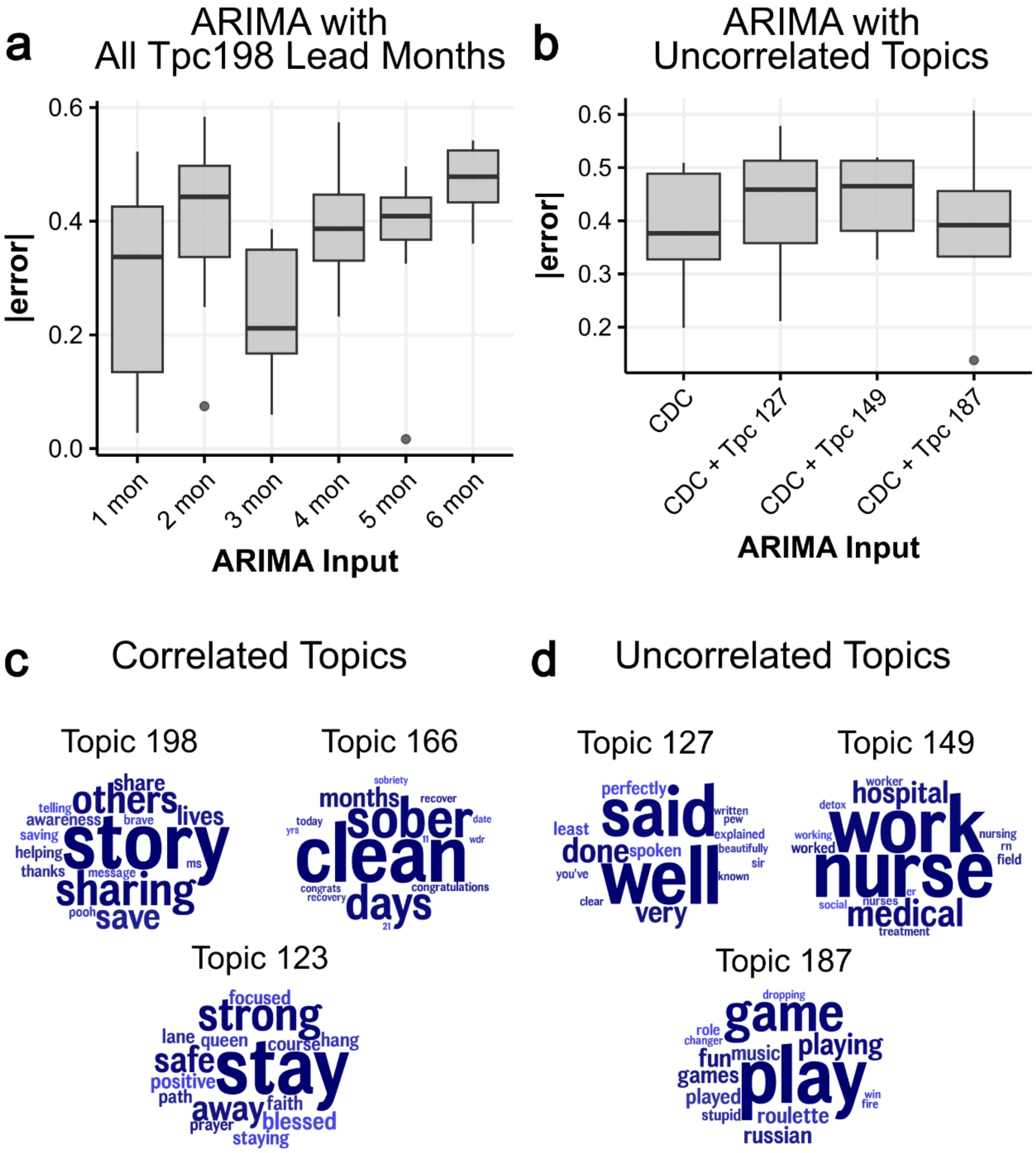

Fig. S3: ARIMA Ablation Results

a. Absolute error distributions for ARIMA models that forecast synthetic opioid overdose death rates. We incorporated different lagged versions of Topic 198 as exogenous variables into ARIMA models. The model trained with a 3-month lagged Topic 198 achieved the lowest mean absolute error.

b. Absolute error distributions for ARIMA models that forecast synthetic opioid overdose death rates. Incorporating uncorrelated topics (topics 127, 149, and 187) as exogenous variables worsened the performance of the ARIMA models.

c,d. Word clouds for topics that improved model performance (correlated) and topics that did not improve model performance (uncorrelated).

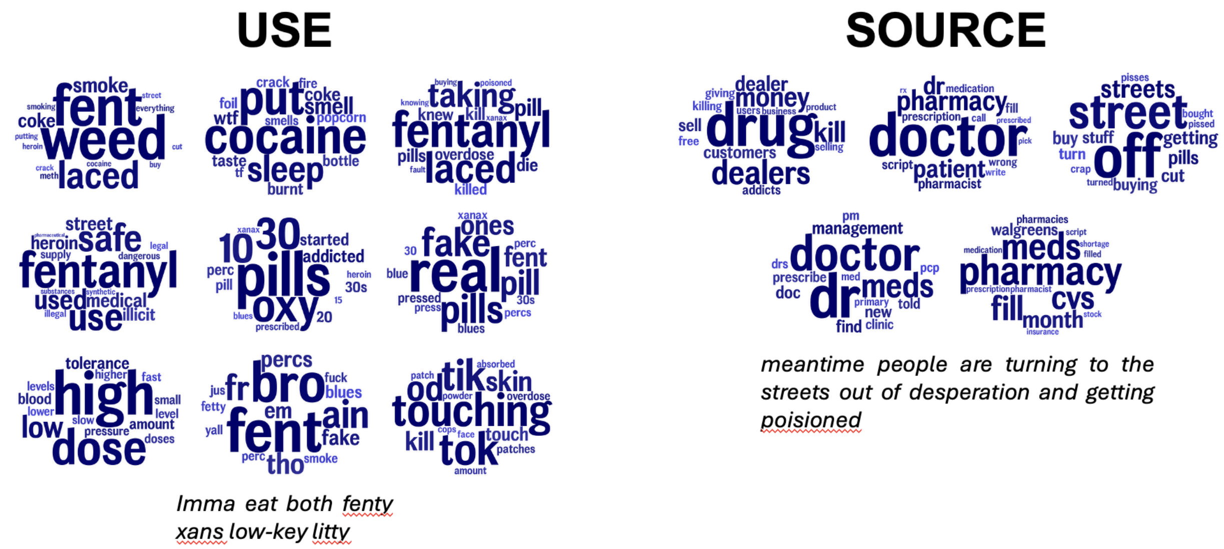

Fig. S4: Use and Source LDA topics

Extra topics under the Active Use category

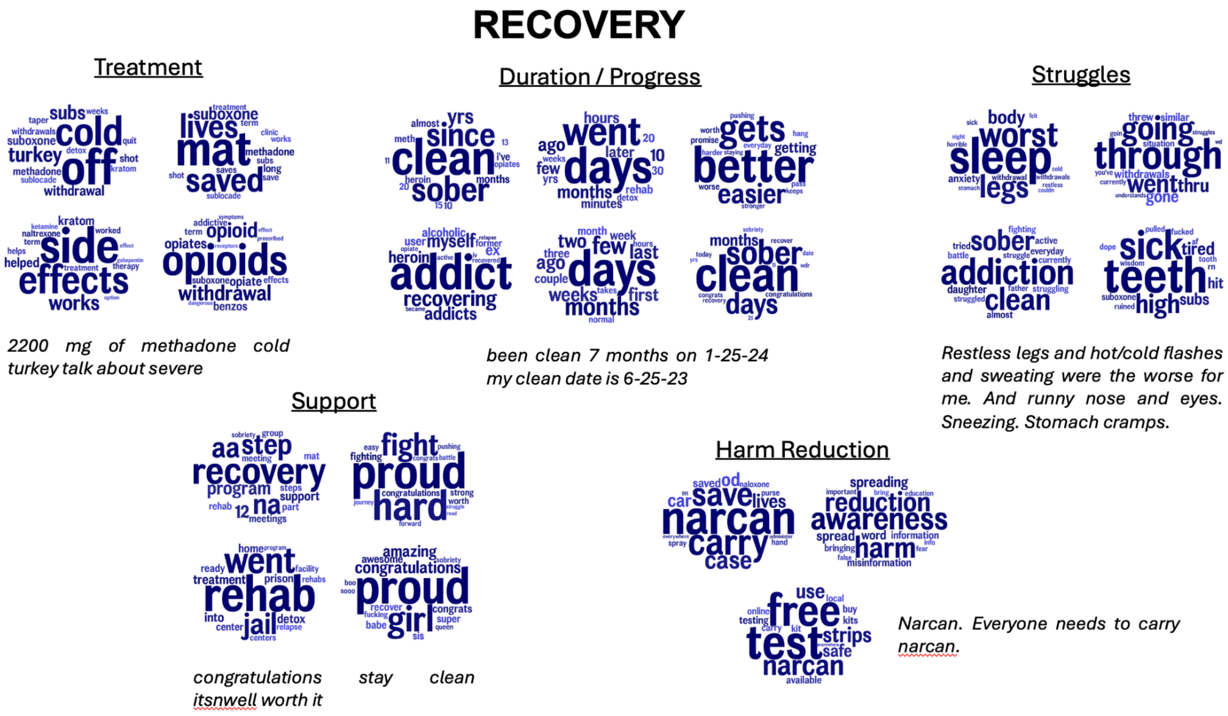

Fig. S5: Recovery and Harm Reduction LDA topics

Extra topics under the Recovery category

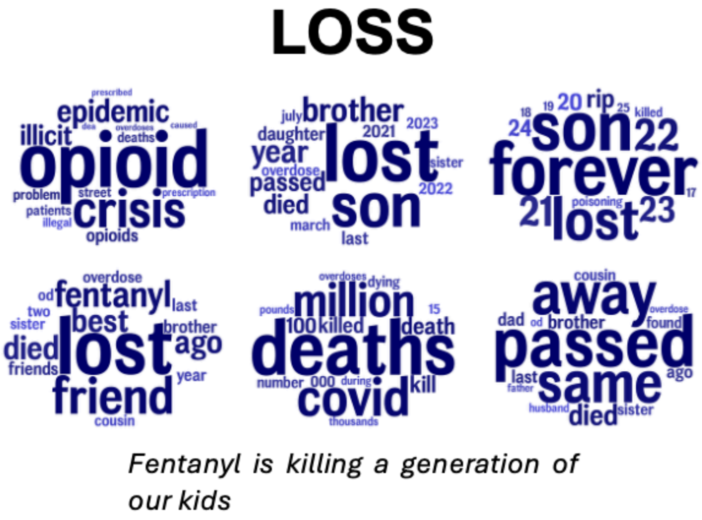

Fig. S6: Loss topics

Extra topics under the Loss category

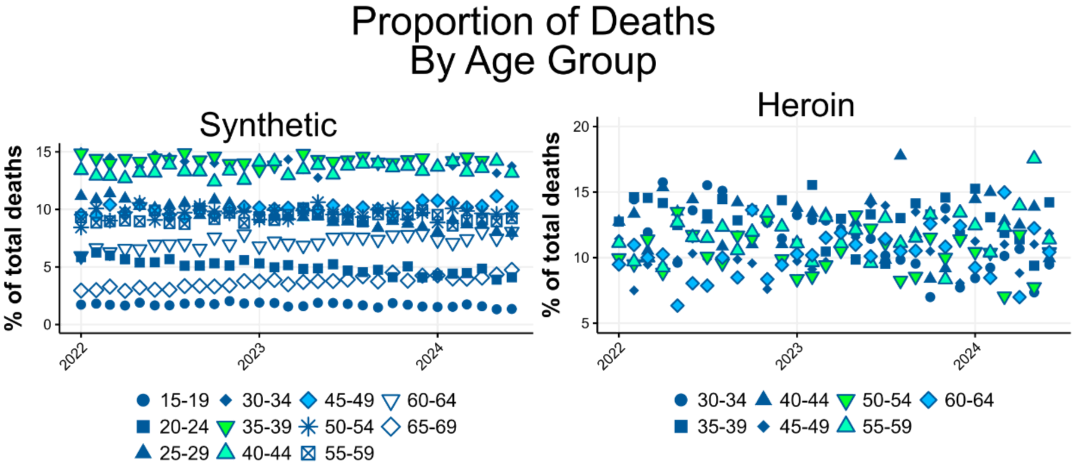

Fig. S7: Mortality Rate By Age Group

a. TikTok topic correlations with opioid overdose death rates categorized by age. For natural semi-synthetic opioids, we do not see any patterning by age.

b. Temporal trend of proportion of opioid overdose deaths by age group

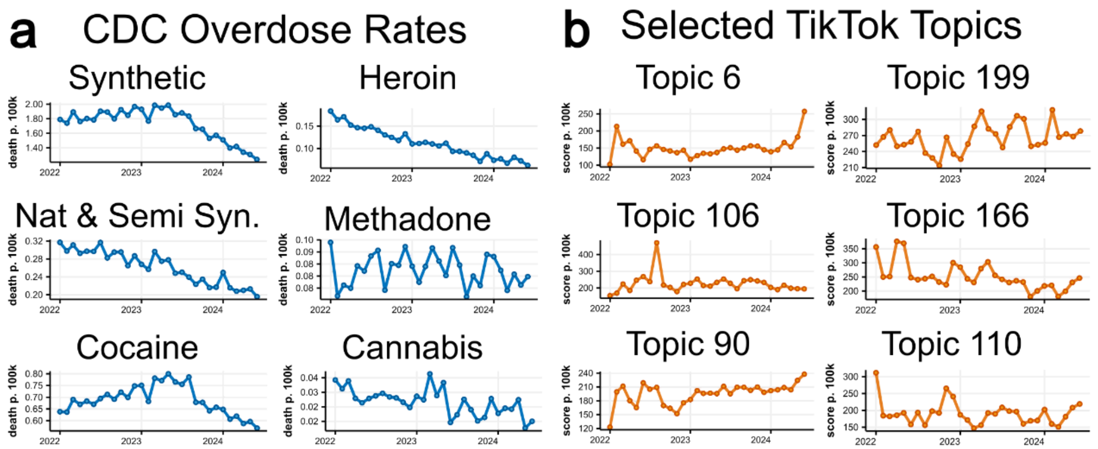

Fig. S8: Time series trends

a. Time series trend of death rates caused by substances classified under the T40 ICD-10 code

b. Time series of selected TikTok topics. Some topics increased over time, others decreased over time, whereas others remained constant over time.

c. TikTok Lead Month Distribution for topics that are positively correlated with CDC opioid overdose death rates.

SUPPLEMENTARY NOTES

Supplementary Note 1: Opioid Keyword List (n = 28 keywords)

“fentanyl”, “opioid”, “opiate”, “morphine”, “codeine”, “oxycodone”, “oxymorphone”, “mscontin”, “percocet”, “sublimaze”, “duragesic”, “fentanil”, “sufentanil”, “fentanylum”, “fentora”, “thebaine”, “codiene”, “roxanol”, “kadian”, “oxycontin”, “roxicodone”, “roxicet”, “endocet”, “endocodone”, “oxyir”, “oxynorm”, “hydrocodone”, “vicodin”

Supplementary Note 2: GPT instruction prompt

I will give you a social media comment (delimited with the xml tags <comment></comment>) to annotate. This comment is part of a larger drugs discussion (e.g., about opioids, etc). Your task is to determine the perspective(s) expressed about drugs—specifically focusing on the main, central drug-related message of the comment. When considering perspectives, take into account not only the presence of pronouns, but more importantly, whether that perspective is central to the comment’s description of drug use, recovery, overdose loss, or other drug-related experiences. Do not automatically register a perspective simply because a pronoun appears; ensure that its use meaningfully contributes to the drug-related context.

You must return a onehot integer of length 3 (e.g., 010) corresponding to the following perspectives:

- Position 1 (first person): Mark 1 if the comment’s main drug-related message comes from the speaker’s own experience or personal account (e.g., “I am recovering from OUD” or “I’ve taken every opiate under the sun”). Otherwise, mark 0.
- Position 2 (second person): Mark 1 if the comment directly addresses another individual about their drug use, experience, or advice using second-person pronouns (e.g., “you”, “your”) and this direct address is central to the drug-related content. If such direct addressing is merely incidental or not the focus of the drug discussion, do not mark this perspective.
- Position 3 (third person): Mark 1 if the comment primarily describes someone else’s or a group’s drug-related experiences using third-person pronouns (e.g., “he”, “she”, “they”) or references, and this outside perspective is central to the comment’s drug story. Avoid marking third person if a pronoun appears only in an aside or incidental remark that is not the main point about drugs.

Guidelines and examples:

- As you analyze the comment, identify which parts form the central drug narrative. Ignore incidental mentions of pronouns that do not contribute to the primary drug-related message.
- Together with your decision, provide a brief explanation that cites why the comment’s main message is associated with a particular perspective and why any incidental pronoun use was not considered central.

For example:

- For “I am recovering from OUD,” return 100 because the drug-related focus is on the speaker’s personal experience.
- For “I am sorry to hear you lost your son to fentanyl OD,” return 010 because the comment directly addresses someone about their drug-related loss.
- For “They take fentanyl-laced substances,” return 001 because the comment describes another group’s drug behavior.
- For “I lost a partner. I am sorry to hear you lost someone too. My condolences to you and your family,” return 110 if both the speaker’s loss and the direct address are central to the drug-related discussion.
- For “You should take a lower dose. They should be careful with supplies from the street,” return 011 if the comment’s main advice and warnings pertain to others and a direct second person.

Important: Focus on the central drug narrative. For example, in the comment below:
"She was there. I have done every dope. Quitting perc is too much hell"
Although the word “She” is used, the dominant, central message is the speaker’s own experience with opiates and drug withdrawal. Therefore, only the first person perspective should be marked (100), and the incidental third-person reference should be ignored.

Now, consider the following comments (delimited with the xml tags <comment></comment>). Please analyze it carefully, decide on the most central drug-related perspective(s), provide a onehot annotation (e.g., 100), and explain your reasoning in detail. Your output should be in the format: "<ann>{your annotation}</ann> <exp>{your reasoning}</exp>\n<ann>{your annotation}</ann> <exp>{your reasoning}</exp>\n...". Response for each comment should be separated by "\n".

Provide your annotation and reasoning for the following comments (delimited with the xml tags <comment></comment>):

Supplementary Note 3: Opioid POV Annotation and GPT Performance

Two authors, IAS and KAC, independently annotated the opioid POV of 500 randomly selected comments. We used Cohen's Kappa to evaluate inter-rater agreement. The Cohen's Kappa values for first, second, and third person POV annotations are 0.75, 0.74, and 0.67, respectively. Per Landis and Koch's popular interpretation[1], these values represent substantial agreement between the annotators. Thus, all analyses were performed using labels from the first annotator (IAS).

Of these 500 comments, 14% were annotated as first-person POV, 6% were annotated as second-person POV, and 14% were annotated as third-person POV. Overall, 29% of comments had at least one of three POVs.

The combination of GPT o1-mini and our prompt achieved 92% sensitivity, 71% specificity, and 82% AUC-ROC in annotating first-person opioid POV, 87% sensitivity, 74% specificity, and 81% AUC-ROC in annotating second-person opioid POV, and 63% sensitivity, 76% specificity, and 70% AUC-ROC third-person opioid POV (Fig. 5c). Overall, the setup had a sensitivity of 80%, a recall of 74%, and AUC-ROC of 77% (micro-averaged).

Supplementary Note 4: List of 75 topics cluster that were positively correlated with T40 Drugs

The list of topics are: 15, 131, 41, 136, 80, 58, 11, 186, 164, 123, 98, 189, 178, 76, 182, 111, 68, 36, 66, 146, 151, 160, 60, 50, 77, 190, 45, 110, 180, 166, 35, 75, 114, 141, 103, 93, 102, 28, 5, 91, 194, 130, 21, 101, 65, 57, 54, 107, 157, 85, 37, 177, 152, 119, 42, 170, 191, 192, 59, 48, 25, 115, 129, 83, 175, 32, 198, 128, 12, 100, 84, 8, 2, 1, 154.

Word clouds for these topics are in Table S1.

Supplementary Note 5: LIWC 2015 Pronoun POV

Fig. 5b shows the density distribution of non-zero relative frequencies for each LIWC 2015 pronoun perspective. The distributions of the three perspectives (I vs YOU vs SHEHE) are comparable, suggesting that there is a fair representation of comments authored from all three pronoun perspectives (1st, 2nd, and 3rd person pronoun perspectives).

Supplementary Note 6: Content Moderation and Informal Keywords

TikTok's community guidelines prohibit explicit and harmful content such as discussions that promote the misuse of drugs while encouraging discussions around recovery from SUD and awareness[2,3]. As a result, users often rely on informal or misspelled terms when discussing illicit topics such as drug use[2,4]. An example is "fentanil" for fentanyl. We included common informal terms in our keyword list to better capture relevant comments.

SUPPLEMENTARY TABLES

Supplementary Table 1: Topic Wordclouds

See next page

Filename	Image	Is Relevant to SUD?
topic_0_wc.png	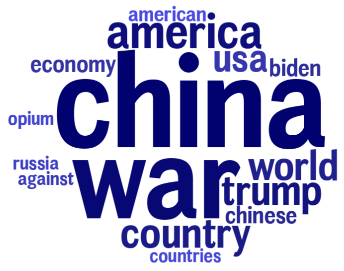	No
topic_1_wc.png	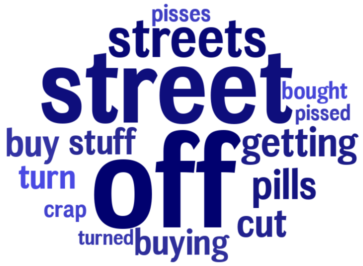	Yes
topic_2_wc.png	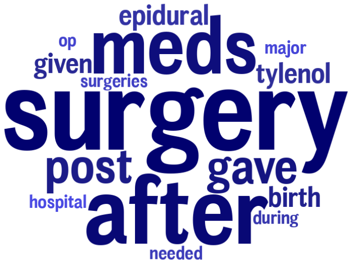	No
topic_3_wc.png	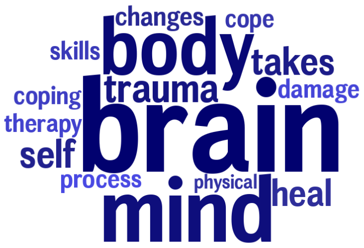	No

topic_4_wc.png	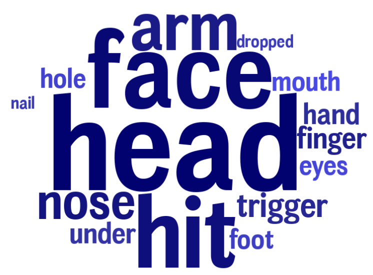	No
topic_5_wc.png	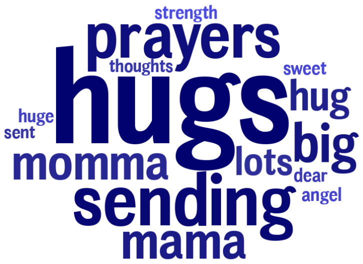	No
topic_6_wc.png	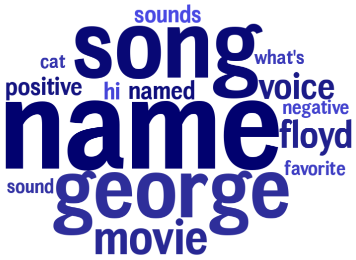	No
topic_7_wc.png	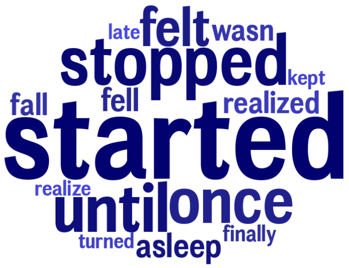	No

<table>
<tr><td>topic_8_wc.png</td><td>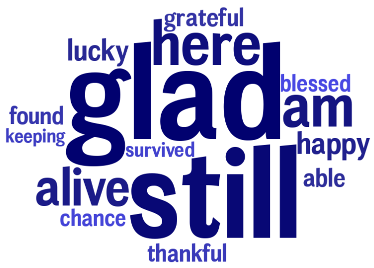
</td><td>No</td></tr>
<tr><td>topic_9_wc.png</td><td>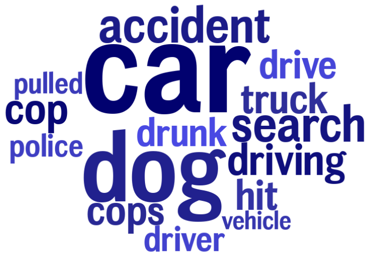
</td><td>No</td></tr>
<tr><td>topic_10_wc.png</td><td>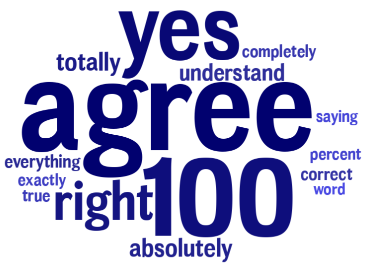
</td><td>No</td></tr>
<tr><td>topic_11_wc.png</td><td>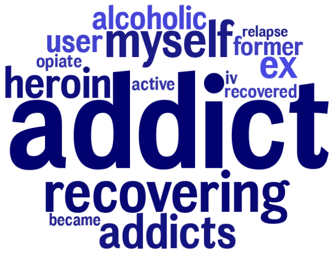
</td><td>Yes</td></tr>
</table>

topic_12_wc.png	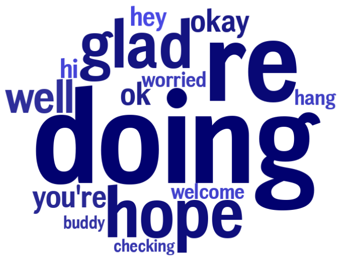	No
topic_13_wc.png	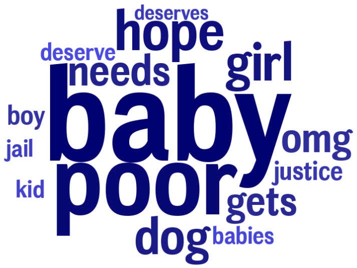	No
topic_14_wc.png	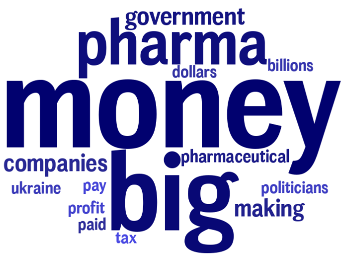	No
topic_15_wc.png	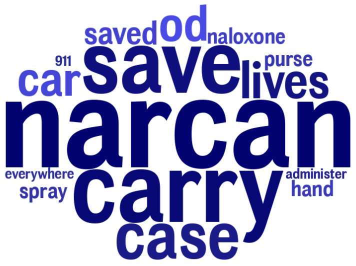	Yes

topic_16_wc.png	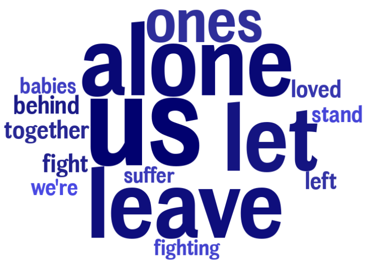	No
topic_17_wc.png	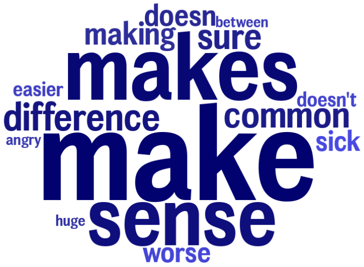	No
topic_18_wc.png	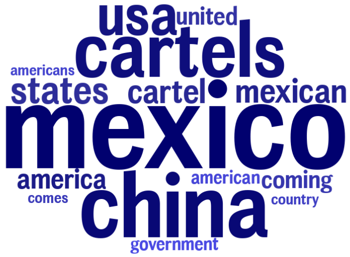	No
topic_19_wc.png	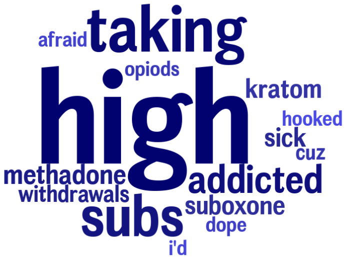	Yes

topic_20_wc.png	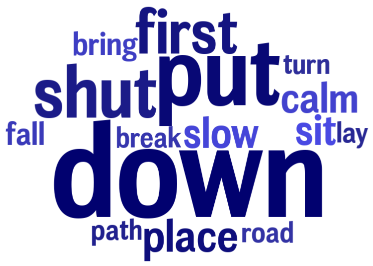	No
topic_21_wc.png	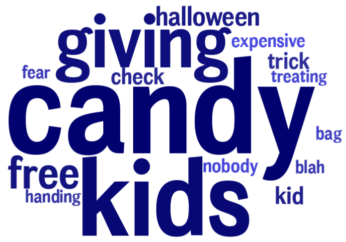	No
topic_22_wc.png	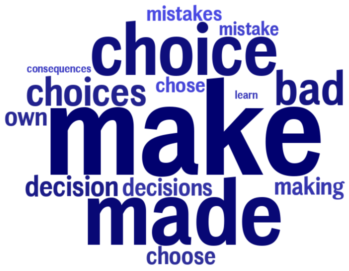	No
topic_23_wc.png	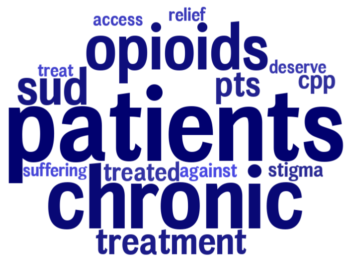	Yes

<table>
<tr><td>topic_24_wc.png</td><td>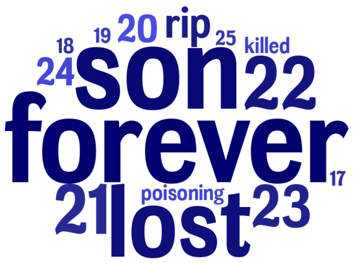
</td><td>Yes</td></tr>
<tr><td>topic_25_wc.png</td><td>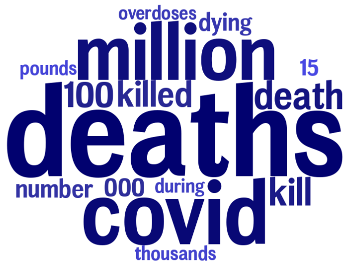
</td><td>Yes</td></tr>
<tr><td>topic_26_wc.png</td><td>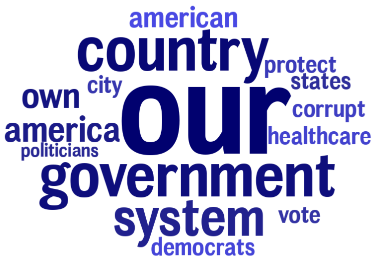
</td><td>No</td></tr>
<tr><td>topic_27_wc.png</td><td>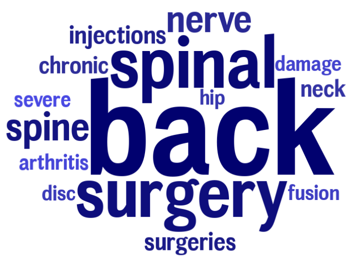
</td><td>No</td></tr>
</table>

topic_28_wc.png	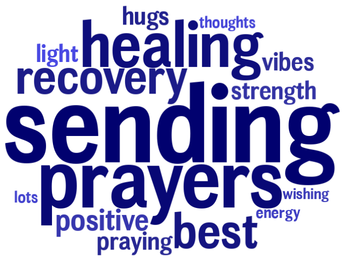	No
topic_29_wc.png	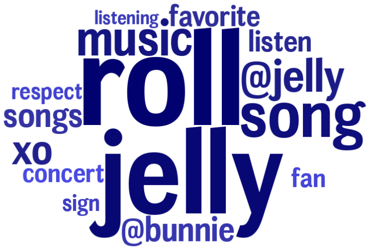	No
topic_30_wc.png	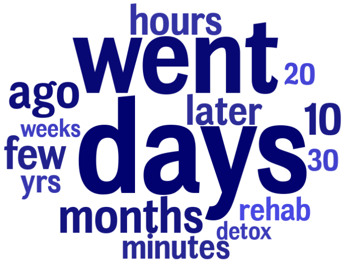	Yes
topic_31_wc.png	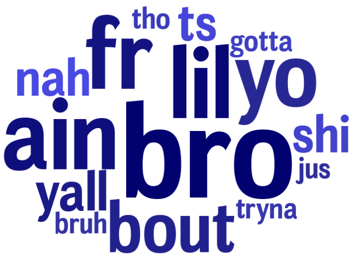	No

topic_32_wc.png	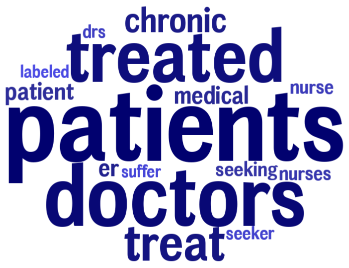	No
topic_33_wc.png	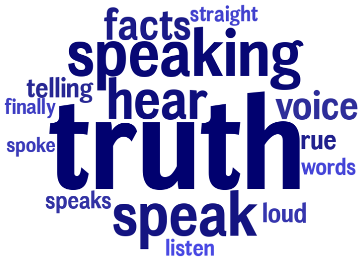	No
topic_34_wc.png	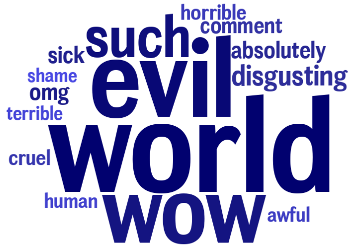	No
topic_35_wc.png	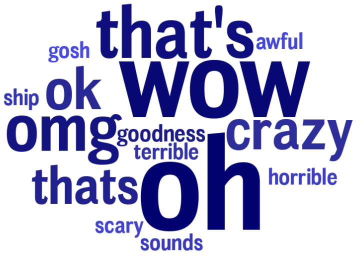	No

topic_36_wc.png	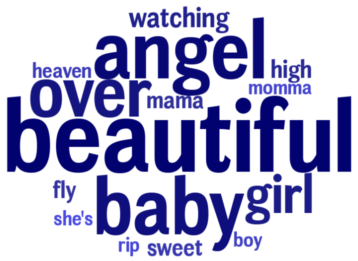	No
topic_37_wc.png	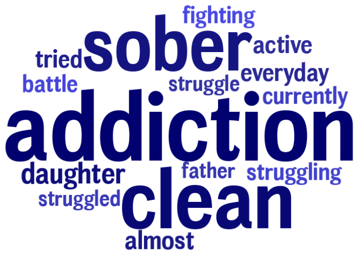	Yes
topic_38_wc.png	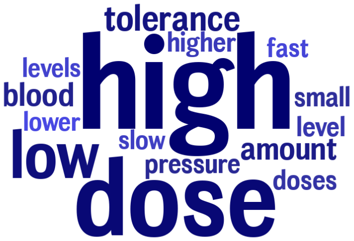	Yes
topic_39_wc.png	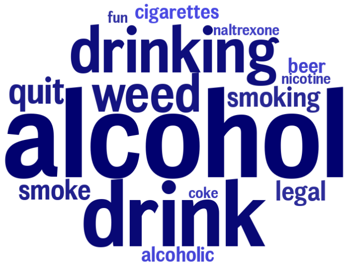	Yes

topic_40_wc.png	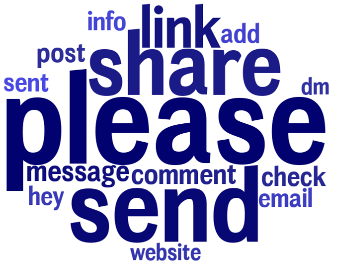	No
topic_41_wc.png	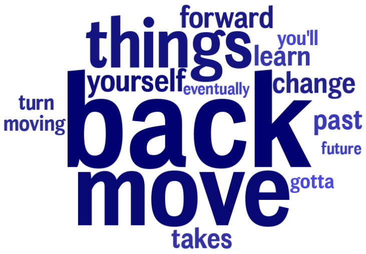	No
topic_42_wc.png	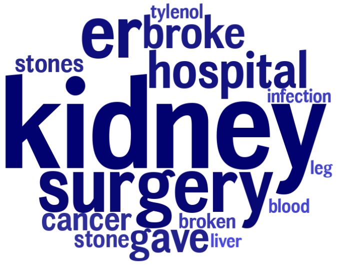	No
topic_43_wc.png	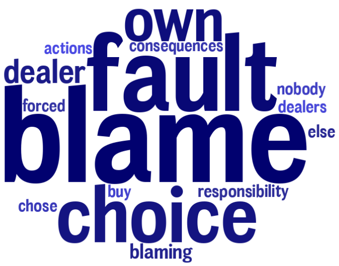	No

topic_44_wc.png	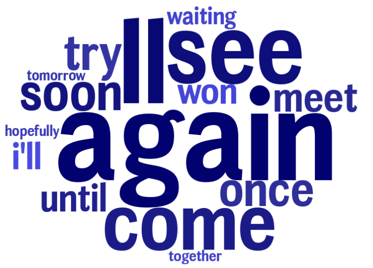	No
topic_45_wc.png	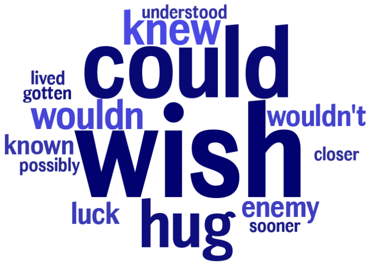	No
topic_46_wc.png	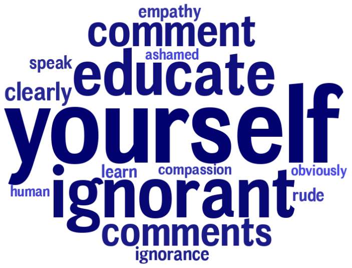	No
topic_47_wc.png	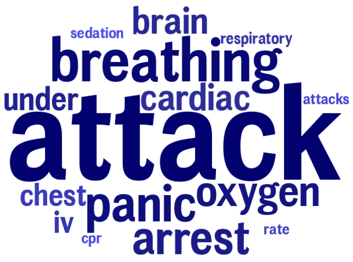	No

topic_48_wc.png	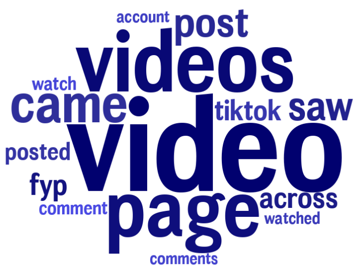	No
topic_49_wc.png	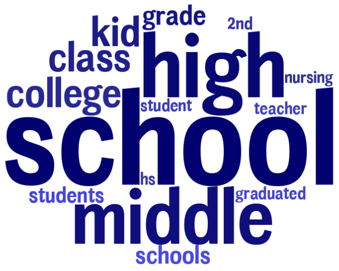	No
topic_50_wc.png	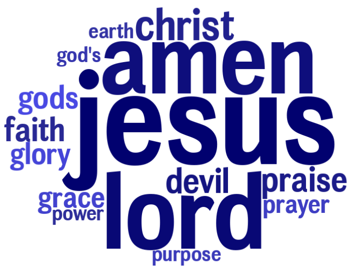	No
topic_51_wc.png	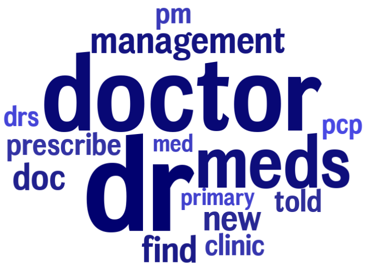	Yes

topic_52_wc.png	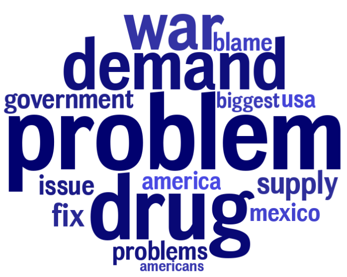	No
topic_53_wc.png	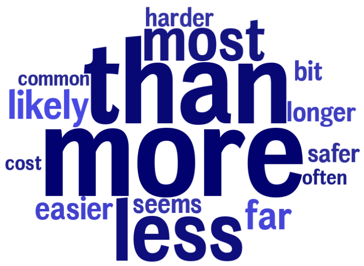	No
topic_54_wc.png	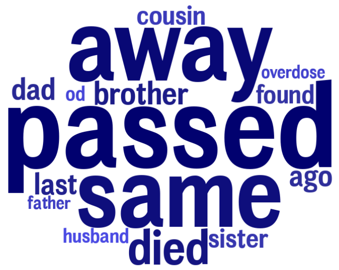	Yes
topic_55_wc.png	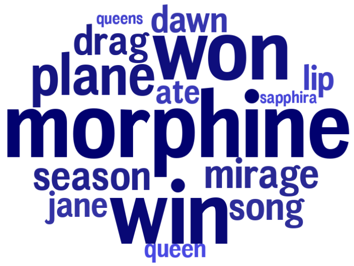	No

<table>
<tr><td>topic_56_wc.png</td><td>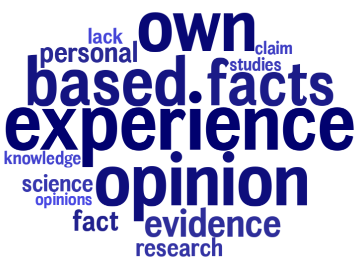
</td><td>No</td></tr>
<tr><td>topic_57_wc.png</td><td>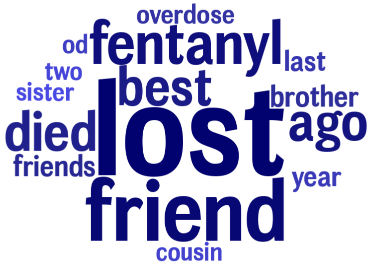
</td><td>Yes</td></tr>
<tr><td>topic_58_wc.png</td><td>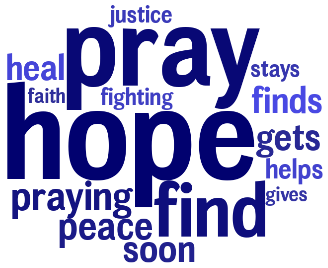
</td><td>No</td></tr>
<tr><td>topic_59_wc.png</td><td>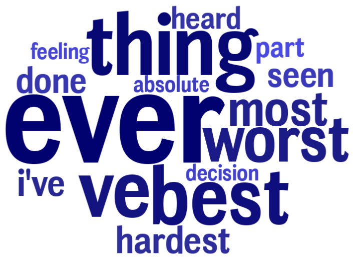
</td><td>No</td></tr>
</table>

topic_60_wc.png	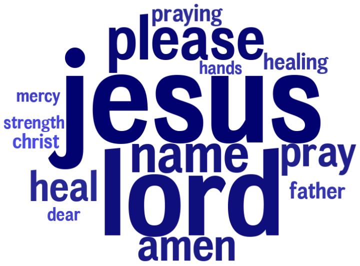	No
topic_61_wc.png	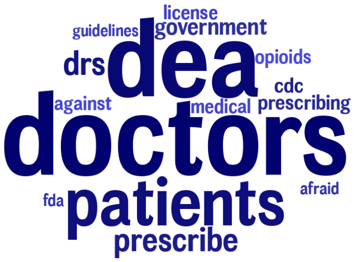	No
topic_62_wc.png	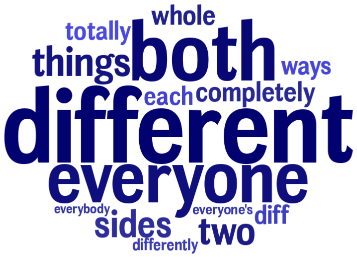	No
topic_63_wc.png	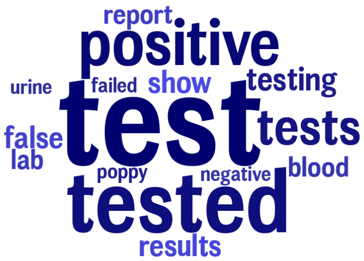	No

topic_64_wc.png	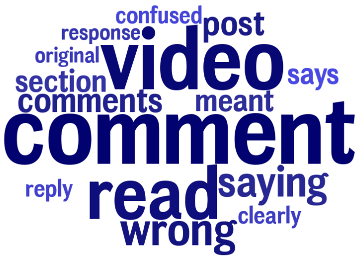	No
topic_65_wc.png	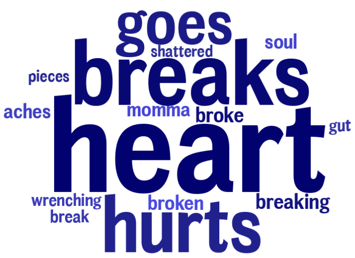	No
topic_66_wc.png	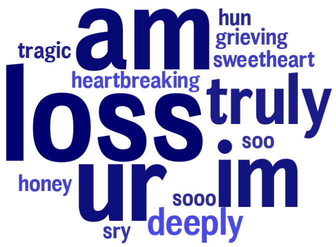	No
topic_67_wc.png	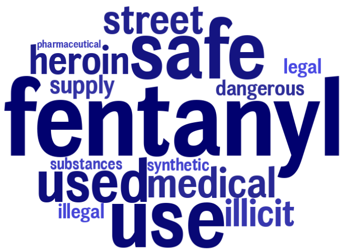	Yes

topic_68_wc.png	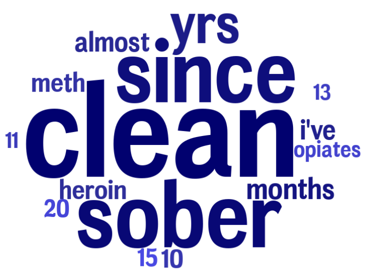	Yes
topic_69_wc.png	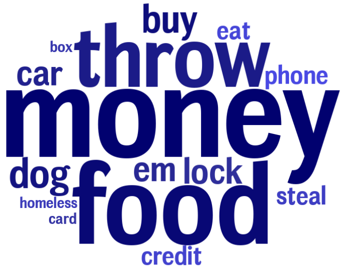	No
topic_70_wc.png	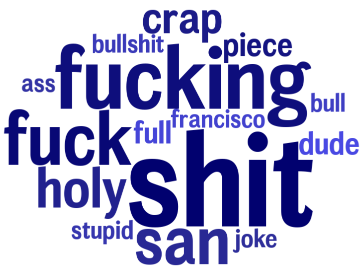	No
topic_71_wc.png	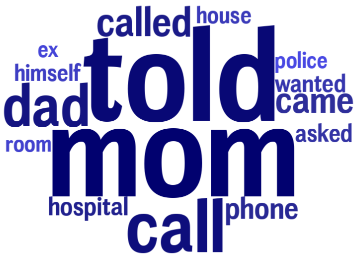	No

topic_72_wc.png	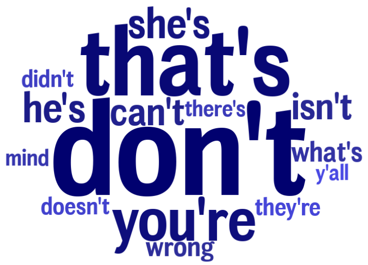	No
topic_73_wc.png	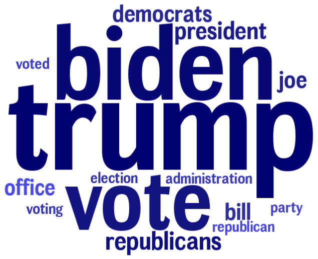	No
topic_74_wc.png	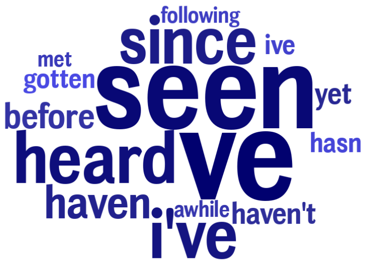	No
topic_75_wc.png	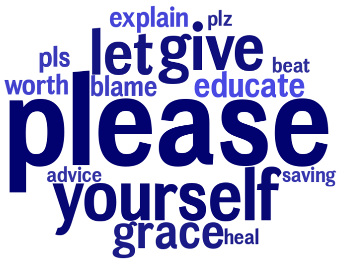	No

topic_76_wc.png	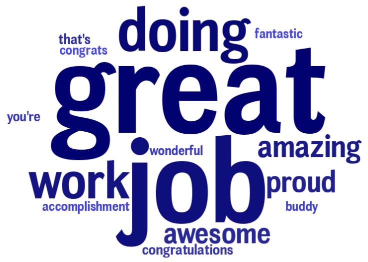	No
topic_77_wc.png	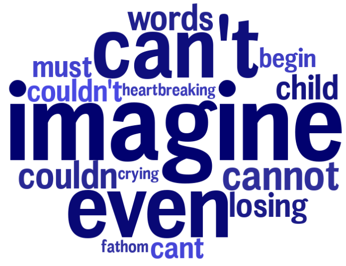	No
topic_78_wc.png	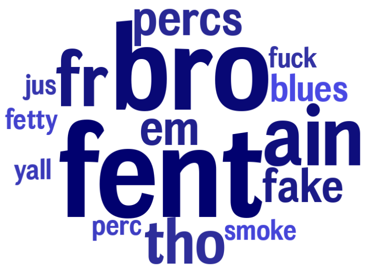	Yes
topic_79_wc.png	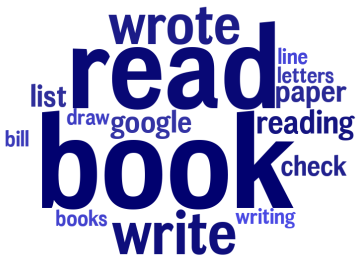	No

topic_80_wc.png	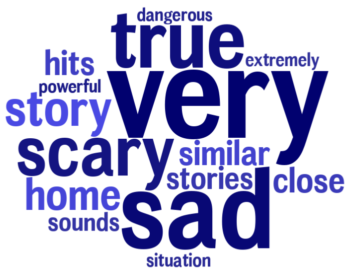	No
topic_81_wc.png	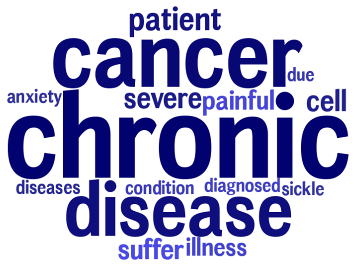	No
topic_82_wc.png	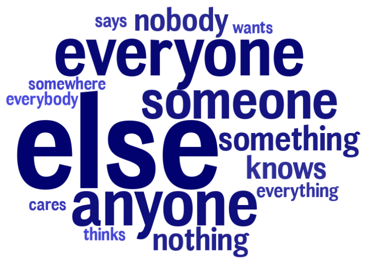	No
topic_83_wc.png	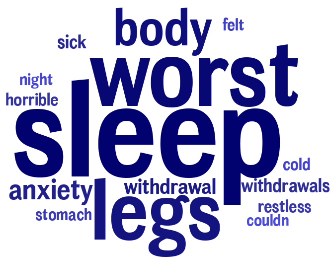	Yes

topic_84_wc.png	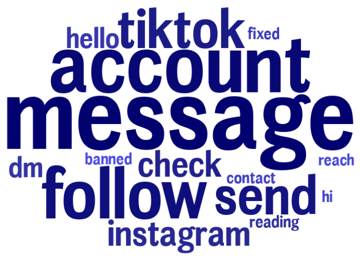	No
topic_85_wc.png	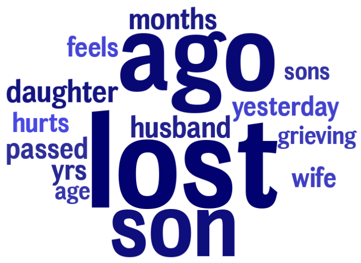	No
topic_86_wc.png	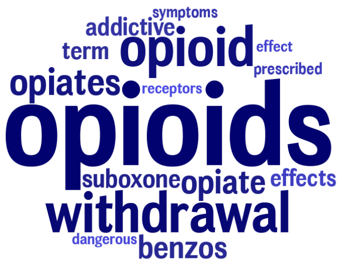	Yes
topic_87_wc.png	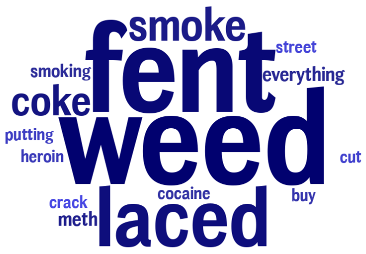	Yes

topic_88_wc.png	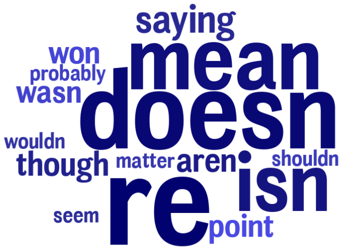	No
topic_89_wc.png	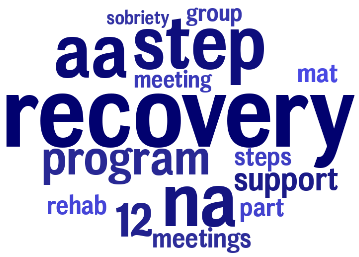	Yes
topic_90_wc.png	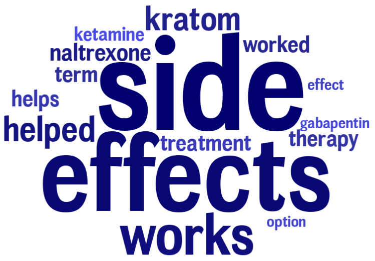	Yes
topic_91_wc.png	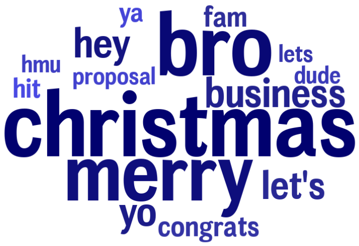	No

<table>
<tr><td>topic_92_wc.png</td><td>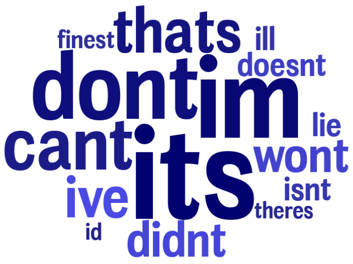
</td><td>No</td></tr>
<tr><td>topic_93_wc.png</td><td>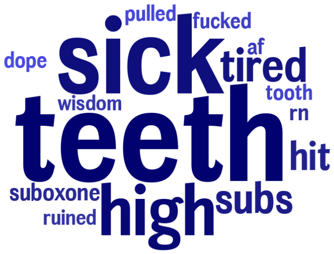
</td><td>Yes</td></tr>
<tr><td>topic_94_wc.png</td><td>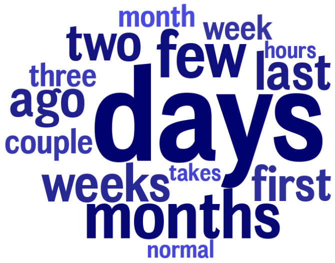
</td><td>Yes</td></tr>
<tr><td>topic_95_wc.png</td><td>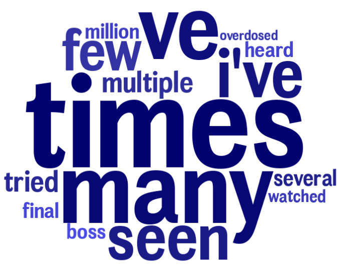
</td><td>No</td></tr>
</table>

topic_96_wc.png	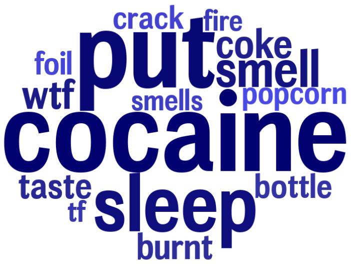	Yes
topic_97_wc.png	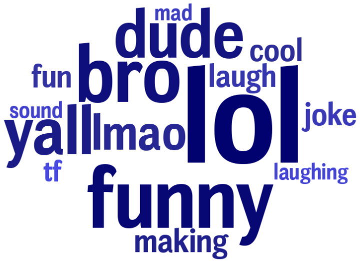	No
topic_98_wc.png	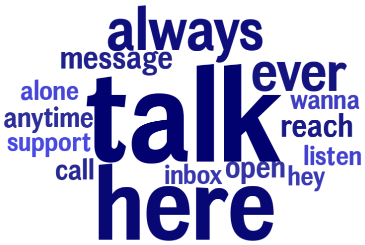	No
topic_99_wc.png	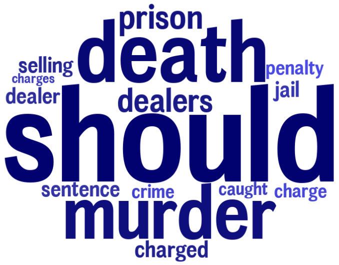	No

<table>
<tr><td>topic_100_wc.png</td><td>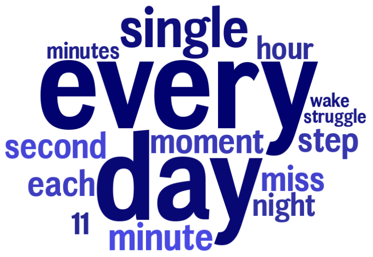
</td><td>No</td></tr>
<tr><td>topic_101_wc.png</td><td>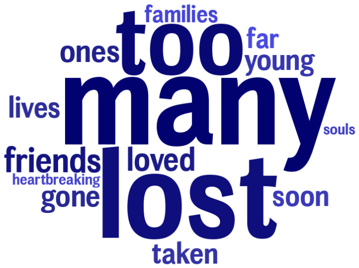
</td><td>No</td></tr>
<tr><td>topic_102_wc.png</td><td>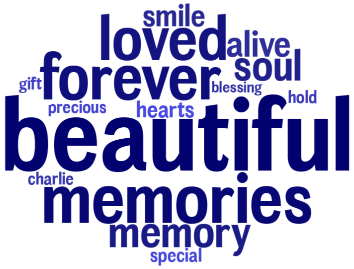
</td><td>No</td></tr>
<tr><td>topic_103_wc.png</td><td>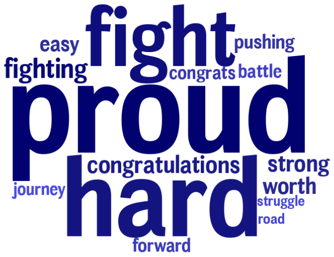
</td><td>No</td></tr>
</table>

topic_104_wc.png	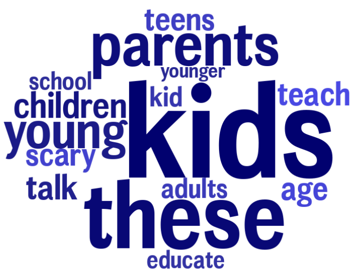	No
topic_105_wc.png	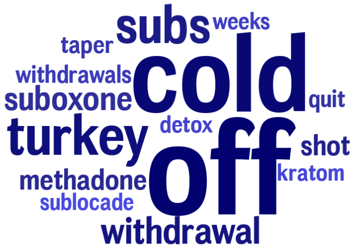	Yes
topic_106_wc.png	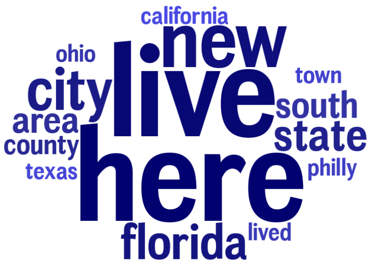	No
topic_107_wc.png	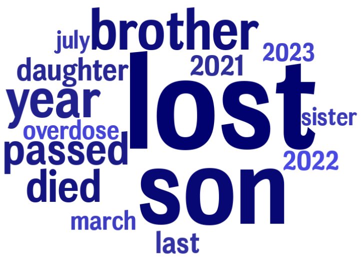	Yes

topic_108_wc.png	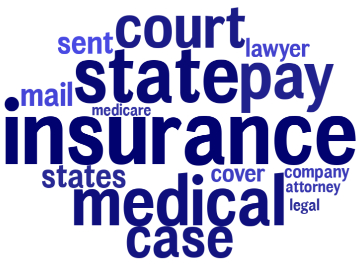	No
topic_109_wc.png	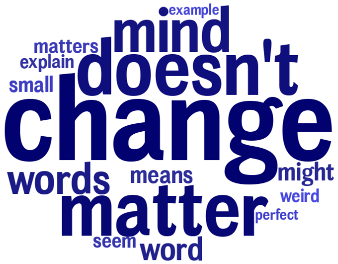	No
topic_110_wc.png	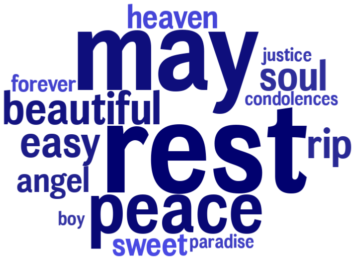	No
topic_111_wc.png	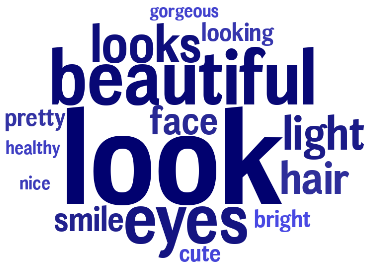	No

<table>
<tr><td>topic_112_wc.png</td><td>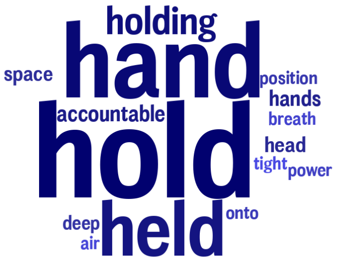
</td><td>No</td></tr>
<tr><td>topic_113_wc.png</td><td>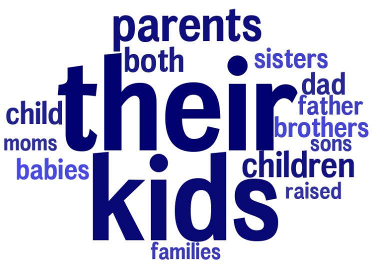
</td><td>No</td></tr>
<tr><td>topic_114_wc.png</td><td>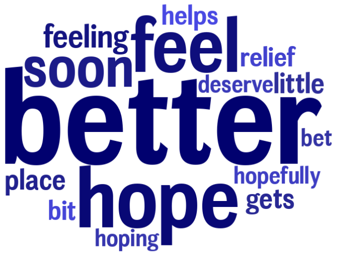
</td><td>No</td></tr>
<tr><td>topic_115_wc.png</td><td>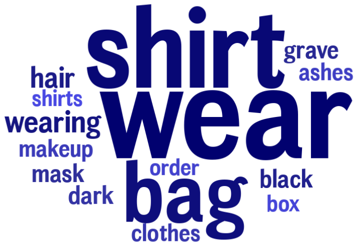
</td><td>No</td></tr>
</table>

topic_116_wc.png	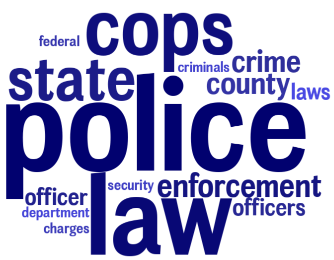	No
topic_117_wc.png	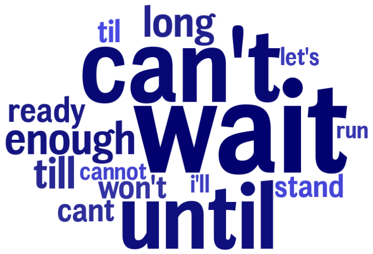	No
topic_118_wc.png	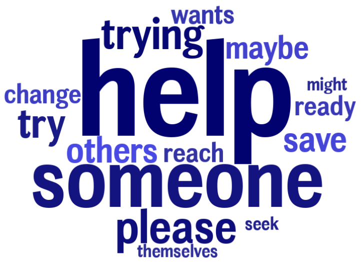	No
topic_119_wc.png	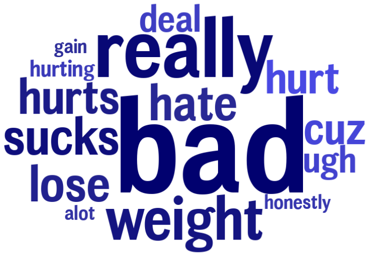	No

topic_120_wc.png	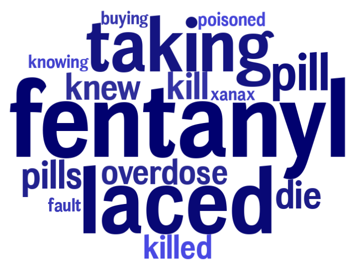	Yes
topic_121_wc.png	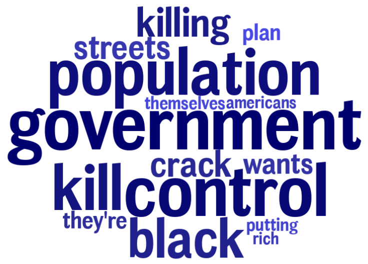	No
topic_122_wc.png	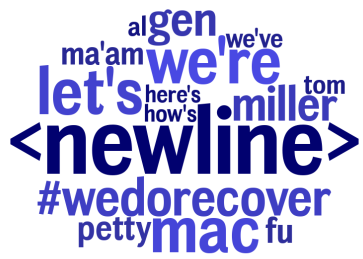	No
topic_123_wc.png	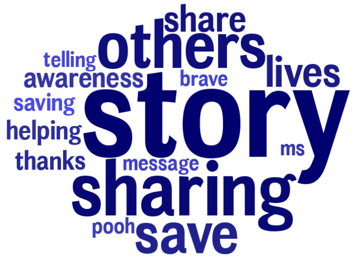	Yes

topic_124_wc.png	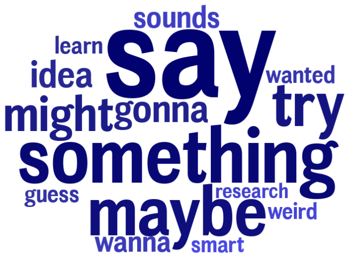	No
topic_125_wc.png	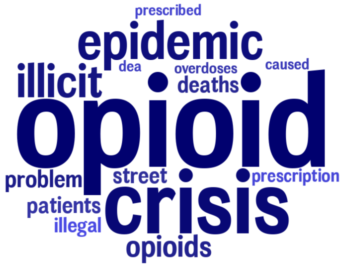	Yes
topic_126_wc.png	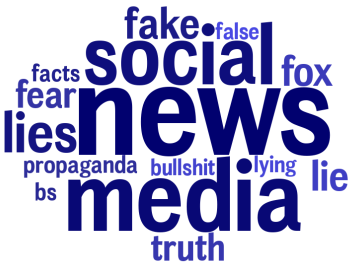	No
topic_127_wc.png	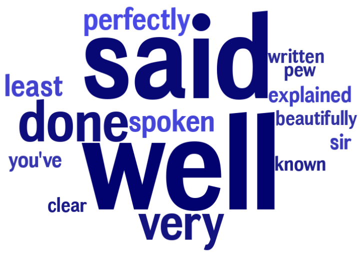	No

topic_128_wc.png	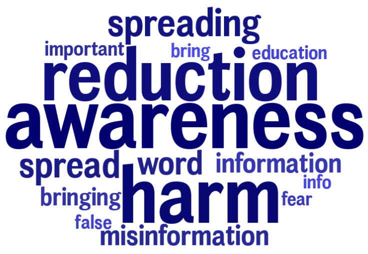	No
topic_129_wc.png	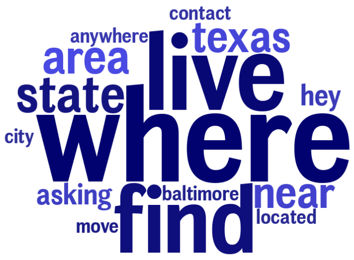	No
topic_130_wc.png	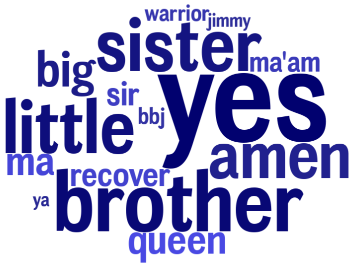	No
topic_131_wc.png	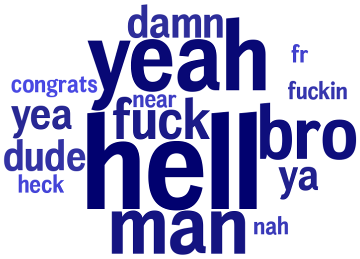	No

topic_132_wc.png	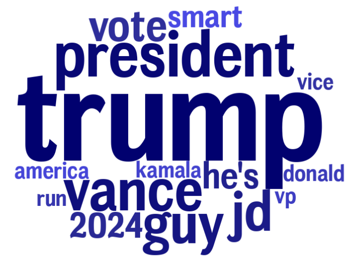	No
topic_133_wc.png	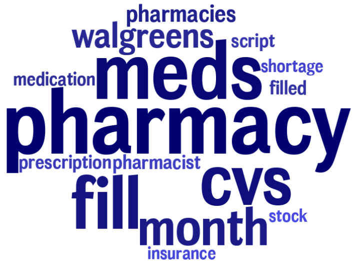	Yes
topic_134_wc.png	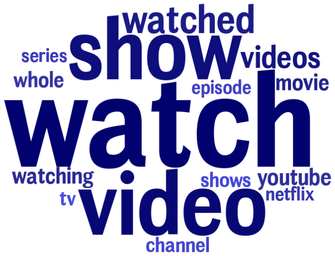	No
topic_135_wc.png	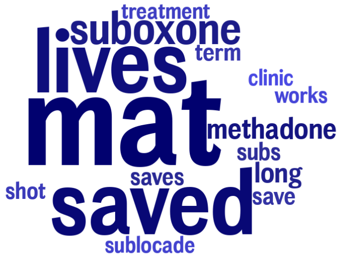	Yes

topic_136_wc.png	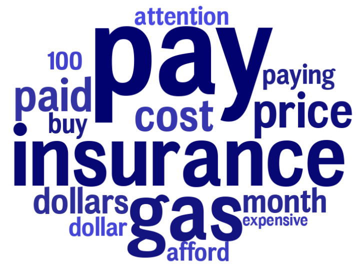	No
topic_137_wc.png	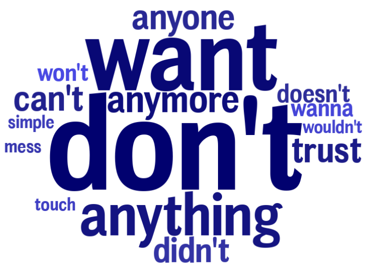	No
topic_138_wc.png	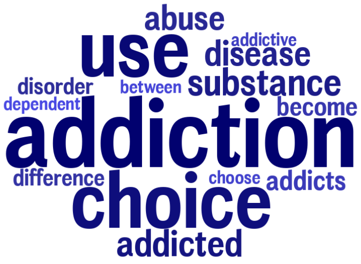	Yes
topic_139_wc.png	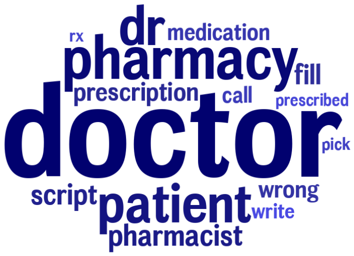	Yes

topic_140_wc.png	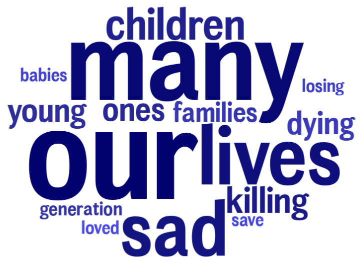	No
topic_141_wc.png	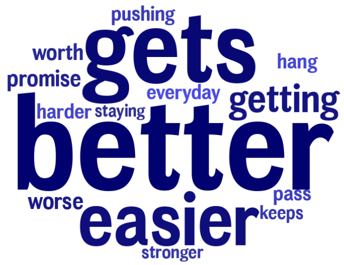	Yes
topic_142_wc.png	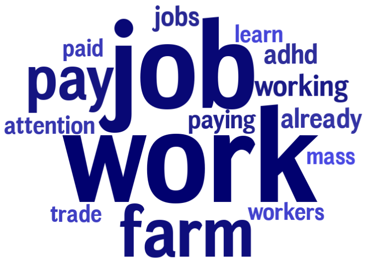	No
topic_143_wc.png	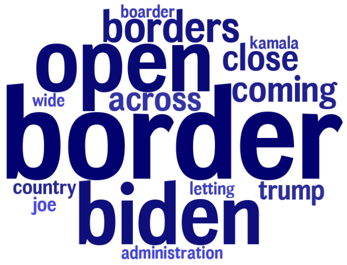	No

topic_144_wc.png	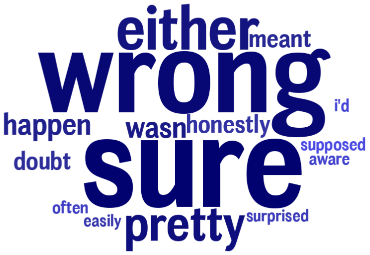	No
topic_145_wc.png	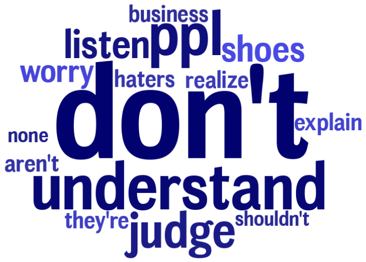	No
topic_146_wc.png	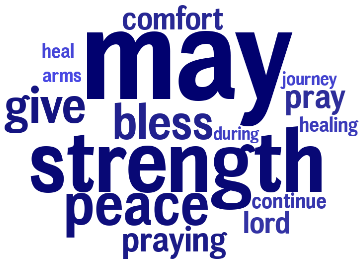	No
topic_147_wc.png	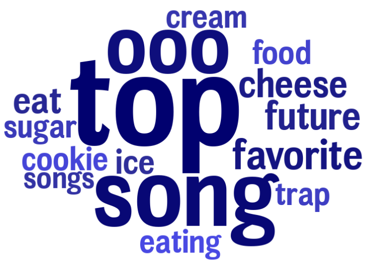	No

<table>
<tr><td>topic_148_wc.png</td><td>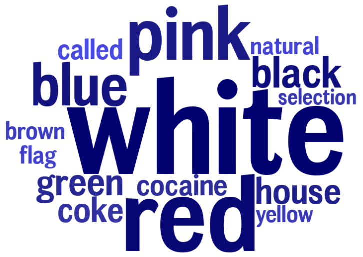
</td><td>No</td></tr>
<tr><td>topic_149_wc.png</td><td>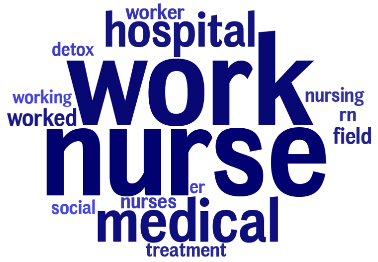
</td><td>No</td></tr>
<tr><td>topic_150_wc.png</td><td>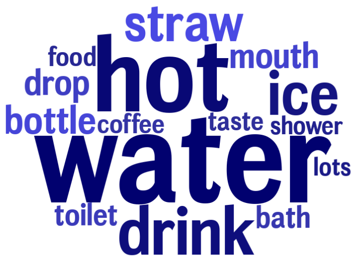
</td><td>No</td></tr>
<tr><td>topic_151_wc.png</td><td>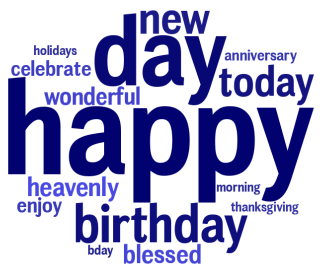
</td><td>No</td></tr>
</table>

topic_152_wc.png	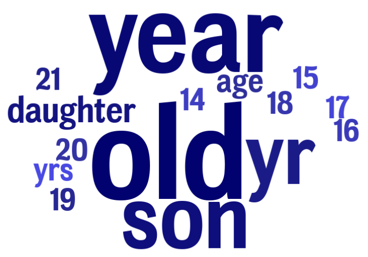	No
topic_153_wc.png	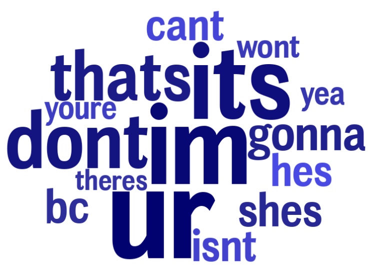	No
topic_154_wc.png	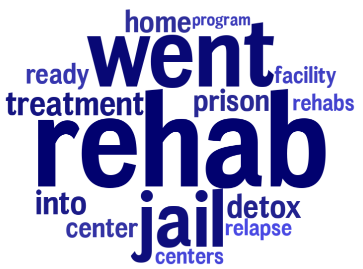	Yes
topic_155_wc.png	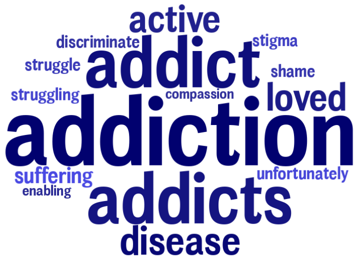	Yes

<table>
<tr><td>topic_156_wc.png</td><td>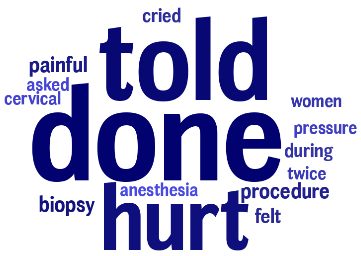
</td><td>No</td></tr>
<tr><td>topic_157_wc.png</td><td>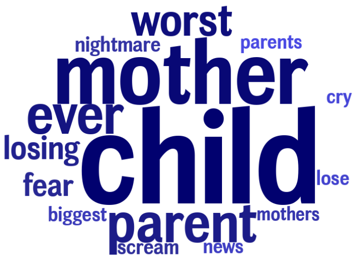
</td><td>No</td></tr>
<tr><td>topic_158_wc.png</td><td>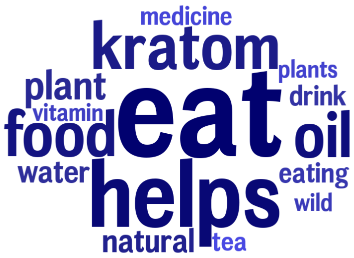
</td><td>No</td></tr>
<tr><td>topic_159_wc.png</td><td>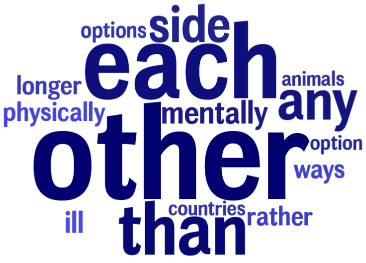
</td><td>No</td></tr>
</table>

topic_160_wc.png	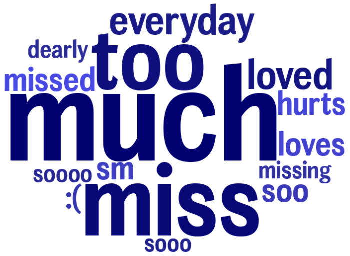	No
topic_161_wc.png	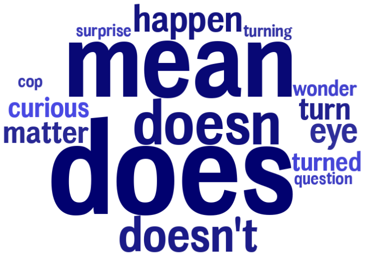	No
topic_162_wc.png	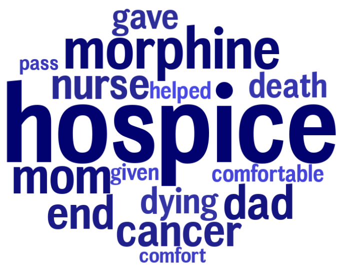	No
topic_163_wc.png	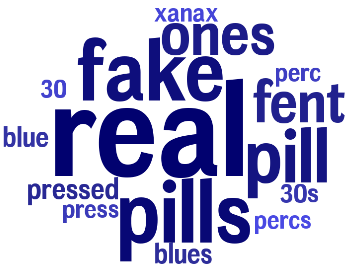	Yes

topic_164_wc.png	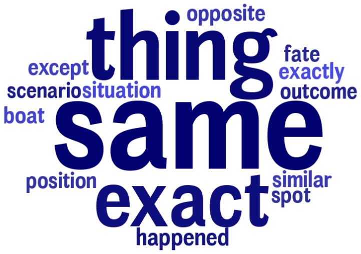	No
topic_165_wc.png	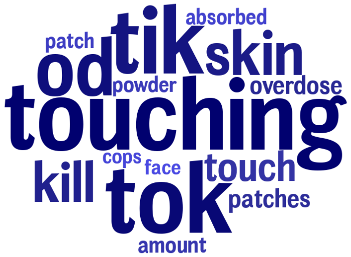	Yes
topic_166_wc.png	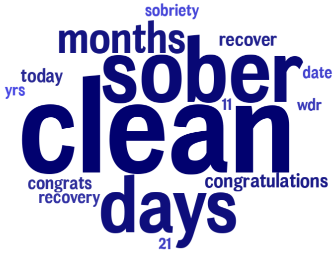	Yes
topic_167_wc.png	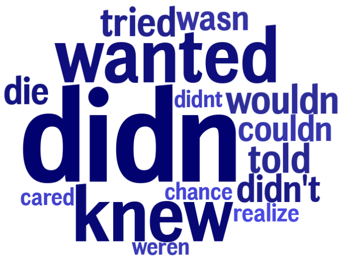	No

topic_168_wc.png	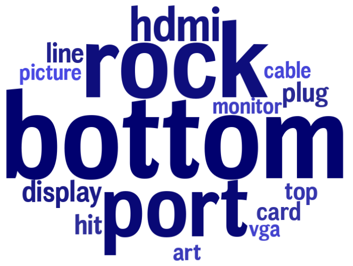	No
topic_169_wc.png	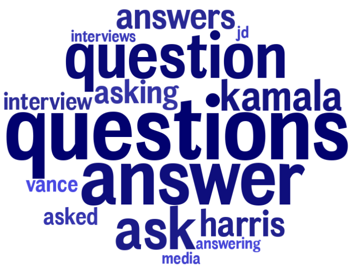	No
topic_170_wc.png	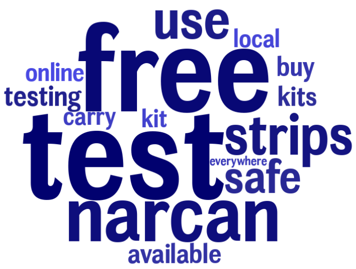	Yes
topic_171_wc.png	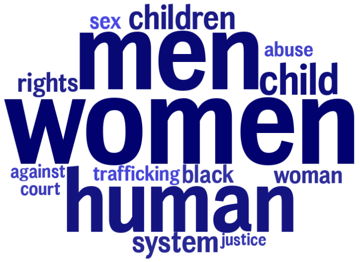	No

topic_172_wc.png	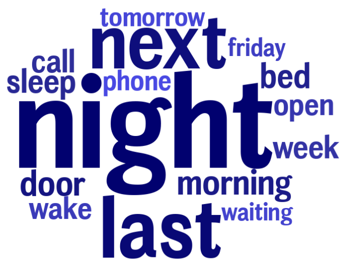	No
topic_173_wc.png	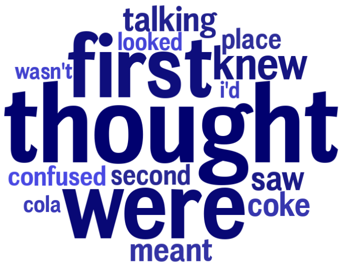	No
topic_174_wc.png	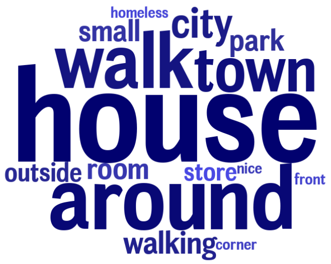	No
topic_175_wc.png	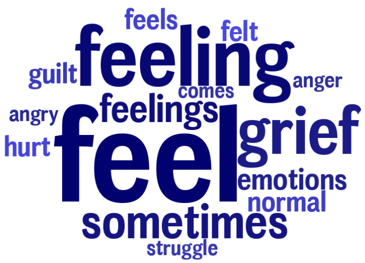	No

topic_176_wc.png	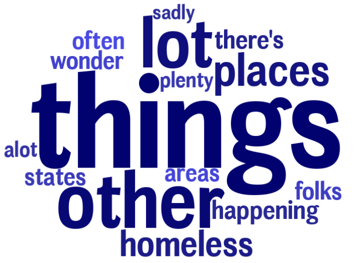	No
topic_177_wc.png	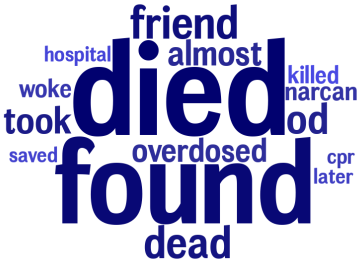	Yes
topic_178_wc.png	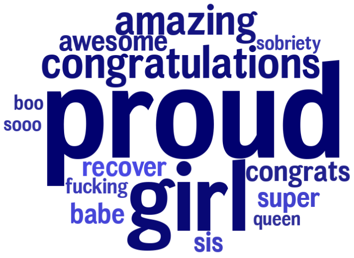	Yes
topic_179_wc.png	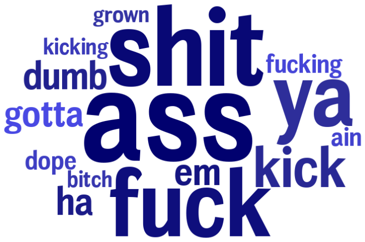	No

topic_180_wc.png	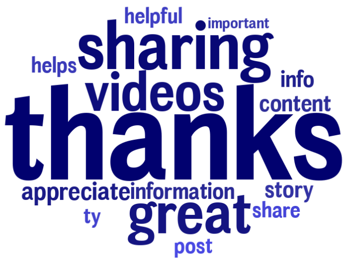	No
topic_181_wc.png	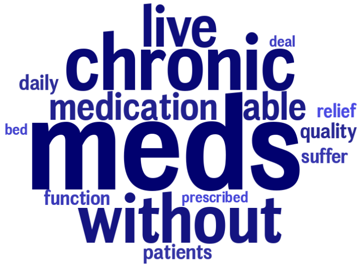	No
topic_182_wc.png	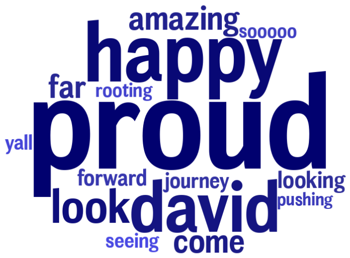	No
topic_183_wc.png	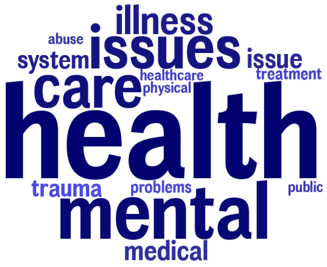	No

topic_184_wc.png	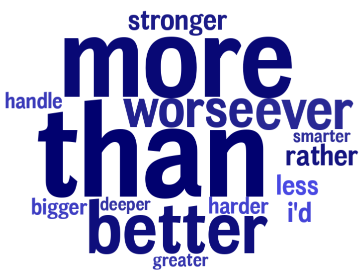	No
topic_185_wc.png	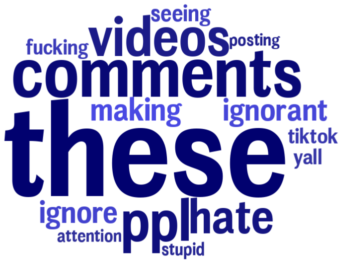	No
topic_186_wc.png	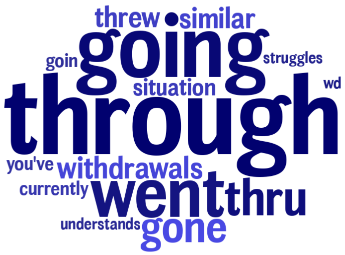	Yes
topic_187_wc.png	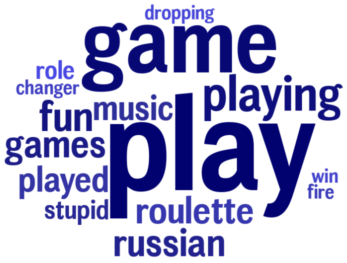	No

topic_188_wc.png	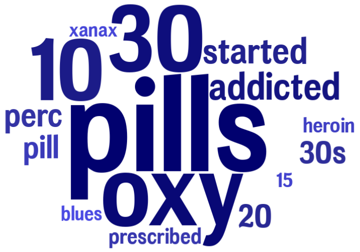	Yes
topic_189_wc.png	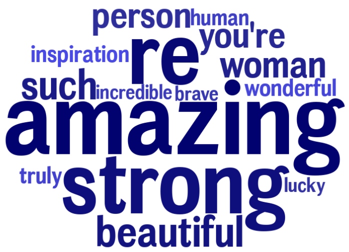	No
topic_190_wc.png	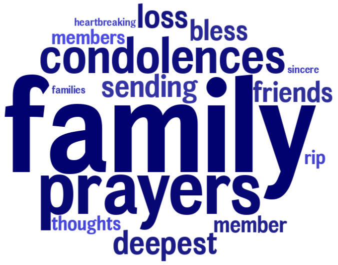	No
topic_191_wc.png	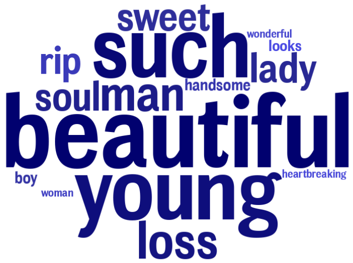	No

topic_192_wc.png	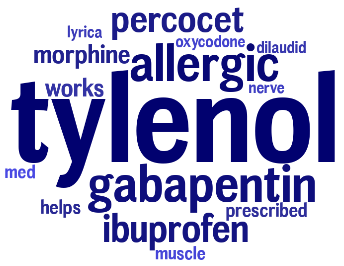	No
topic_193_wc.png	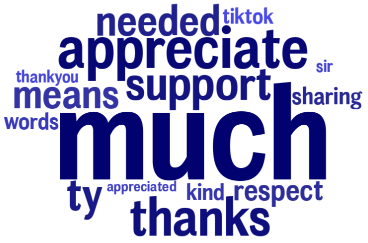	No
topic_194_wc.png	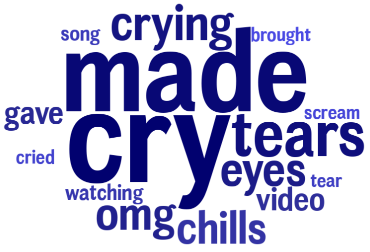	No
topic_195_wc.png	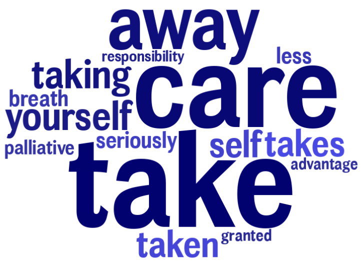	No

<table>
<tr><td>topic_196_wc.png</td><td>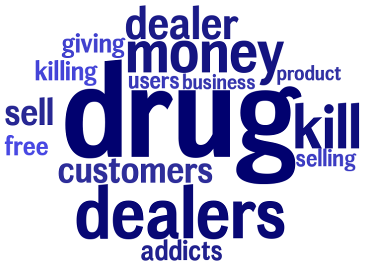
</td><td>Yes</td></tr>
<tr><td>topic_197_wc.png</td><td>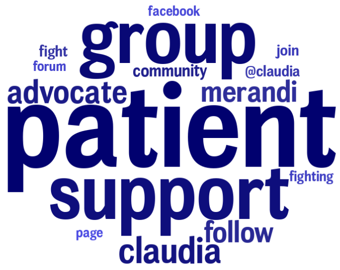
</td><td>No</td></tr>
<tr><td>topic_198_wc.png</td><td>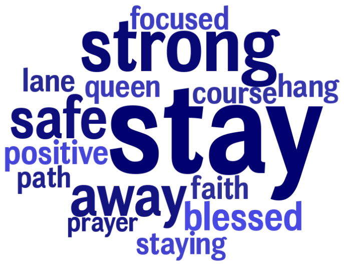
</td><td>No</td></tr>
<tr><td>topic_199_wc.png</td><td>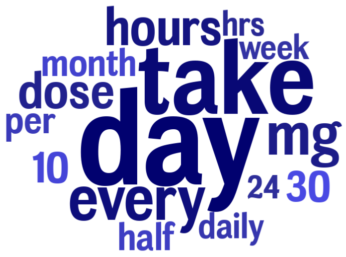
</td><td>Yes</td></tr>
</table>

Supplementary Table 2: Topic Grouping

See next page

Filename	Image					Drug Category	Counts	Proportion (%)
topic_1_wc.png	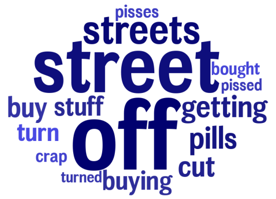	Source				Use Counts	11	23
topic_11_wc.png	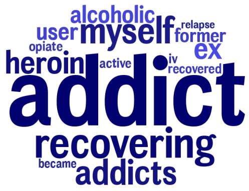	Recovery	Progress			Source Counts	5	11
topic_15_wc.png	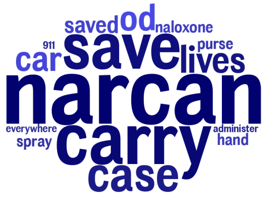	Harm Reduction	Harm Reduction			Recovery Counts	20	43
topic_19_wc.png	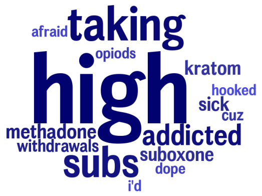	Recovery	Treatment			Loss Counts	7	15
topic_23_wc.png	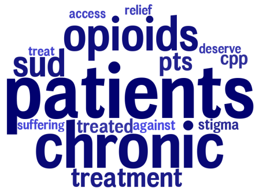	Recovery				Harm Reduction Counts	4	9

topic_24_wc.png	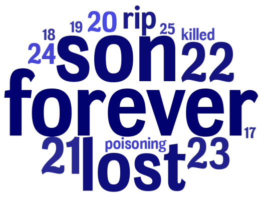	Loss							
topic_25_wc.png	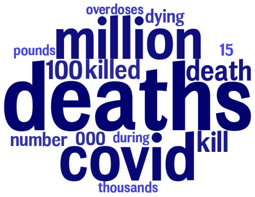	Loss							
topic_30_wc.png	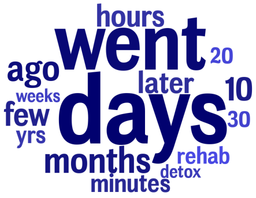	Recovery	Progress						
topic_37_wc.png	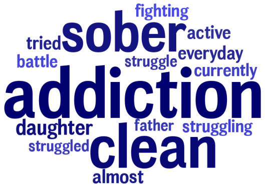	Recovery	Struggles						
topic_38_wc.png	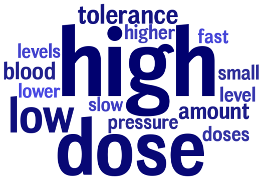	Use							

topic_39_wc.png	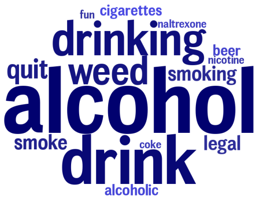	Use							
topic_51_wc.png	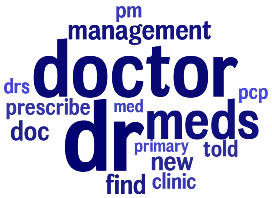	Source							
topic_54_wc.png	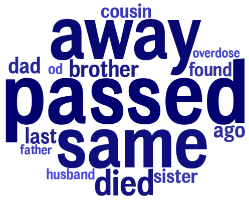	Loss							
topic_57_wc.png	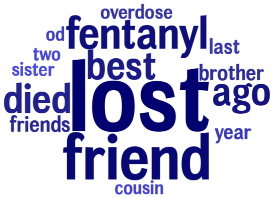	Loss							
topic_67_wc.png	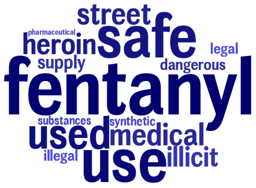	Use							

topic_68_wc.png	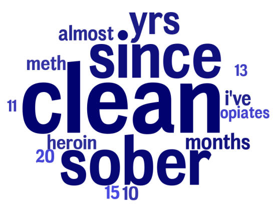	Recovery	Progress						
topic_78_wc.png	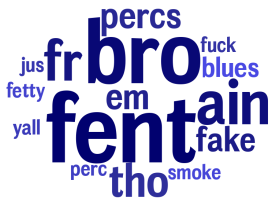	Use							
topic_83_wc.png	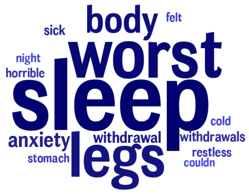	Recovery	Struggles						
topic_86_wc.png	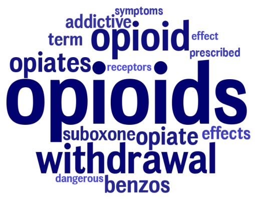	Recovery	Treatment						
topic_87_wc.png	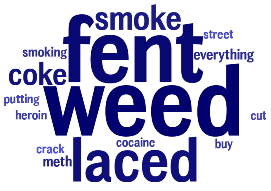	Use							

topic_89_wc.png	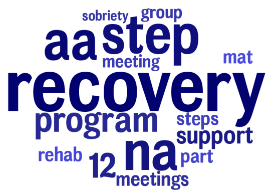	Recovery	Support						
topic_90_wc.png	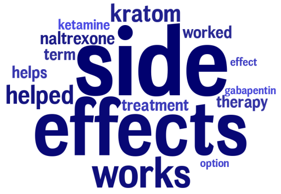	Recovery	Treatment						
topic_93_wc.png	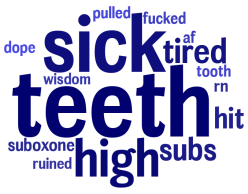	Recovery	Struggles						
topic_94_wc.png	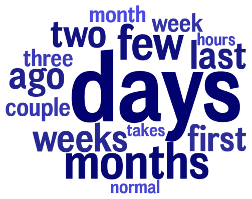	Recovery	Progress						
topic_96_wc.png	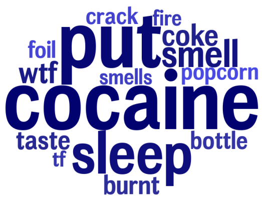	Use							

topic_105_wc.png	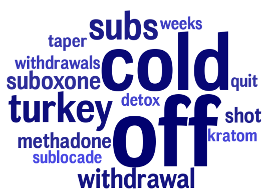	Recovery	Treatment					
topic_107_wc.png	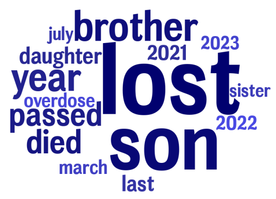	Loss						
topic_120_wc.png	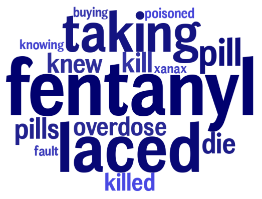	Use						
topic_123_wc.png	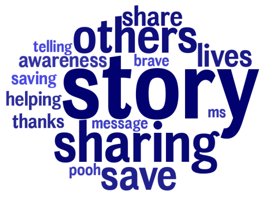	Harm Reduction	Harm Reduction					
topic_125_wc.png	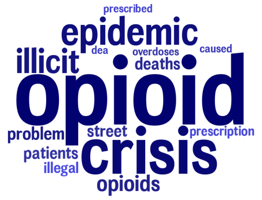	Loss						

topic_133_wc.png	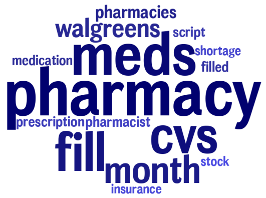	Source							
topic_135_wc.png	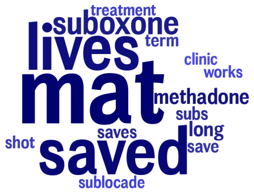	Recovery	Treatment						
topic_138_wc.png	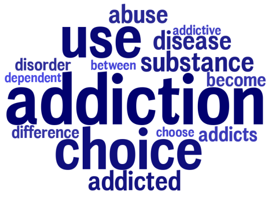	Harm Reduction	Harm Reduction						
topic_139_wc.png	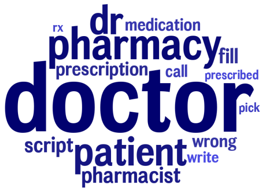	Source							
topic_141_wc.png	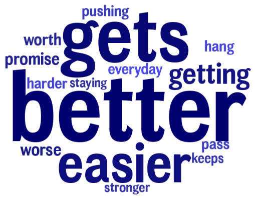	Recovery	Progress						

topic_154_wc.png	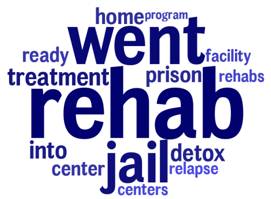	Recovery	Support					
topic_155_wc.png	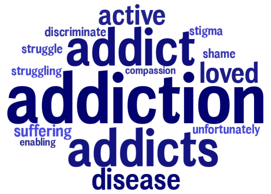	Recovery	Struggles					
topic_163_wc.png	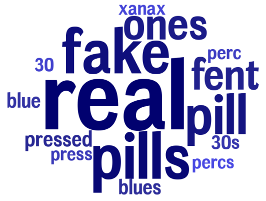	Use						
topic_165_wc.png	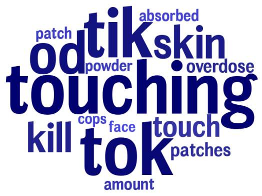	Use						
topic_166_wc.png	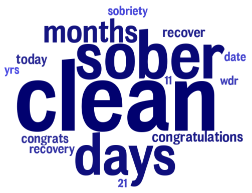	Recovery	Progress					

topic_170_wc.png	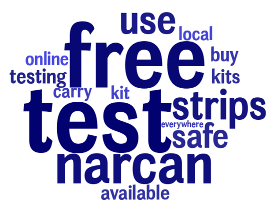	Harm Reduction	Harm Reduction						
topic_177_wc.png	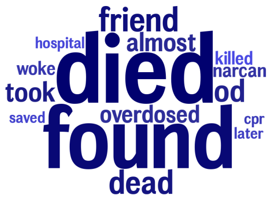	Loss							
topic_178_wc.png	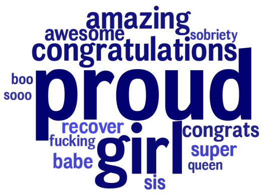	Recovery	Support						
topic_186_wc.png	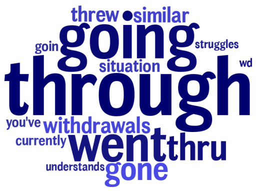	Recovery	Struggles						
topic_188_wc.png	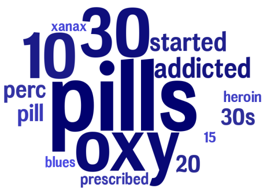	Use							

topic_196_wc.png	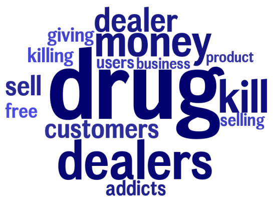	Source							
topic_199_wc.png	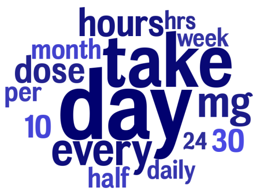	Use							

Supplementary Table 3: Examples of comments and their corresponding opioid perspective annotations assigned by GPT o1-mini. Due to copyright restrictions, emojis were replaced with *<emoji>* tags

A value of 1 in position one, two, or three of the annotation indicates the presence of a first-person, second-person, or third-person opioid perspective, respectively, while 0 indicates otherwise. For example, "100" represents the presence of only first-person opioid perspective, whereas "110" represents the presence of both first-person and second-person opioid perspectives.

Comment	GPT Annot	GPT Reason	General Notes
On judgement day god gon see this comment just saying	000	The comment does not centralize any drug-related perspective from the speaker, another person, or third party. It appears to be a general statement without focusing on personal or others' drug experiences.	
Rip to my number one fent buyer<emoji><emoji>	001	The comment refers to a third party (""my number one fent buyer""), describing someone else's involvement with drugs. This establishes a third-person perspective as the central drug-related message.	"fent" is the slang name for fentanyl
you're probably on perk 10s or something however "perk 30s" aren't real anymore	010	The comment directly addresses another person regarding their likely use of ""perk 10s,"" employing second-person pronouns (""you""), making this perspective central to the drug-related content.	"perk 10" refers to Percocet 10 mg tablets. "perk 30" refers Percocet 30 mg
WD symptoms for me off this shit is yawning, loss of appetite, digestive issues like diarrhea and nausea, mental changes, cravings, anxiety, runny	100	The comment details the speaker's own withdrawal symptoms from drug use, focusing on personal experience.	"WD" means withdrawal
I have been off all substance since 2017. you can recover! I am rooting for you!	110	The comment shares the speaker's personal recovery experience ("I have been off all substance since 2017") and directly encourages someone else's recovery ("you can recover! I am rooting for you!").	
At least in NY addicts are considered a vulnerable population. At my treatment facility we encourage family support.	101	The comment includes the speaker's perspective from their treatment facility (first person) and also refers to addicts as a vulnerable population (third person)	

Ugh. More like dear junkie fucks. Don't do drugs off the street.	011	The comment provides direct advice to others to avoid street drugs (second person) and also describes others as 'junkie fucks'	

Supplementary Table 4: Comments manually annotated with opioid perspective for evaluating GPT o1-mini performance. Due to copyright restrictions, emojis were replaced with <*emoji*> tags.

Comment	Annotation (Manual)
I broke 4 ribs,I was given 30 TylenolW/Cod. It did absolutely nothing for pain. Ibuprofen gave me a tiny bit of relief.i was surprised.	100
He isn't wrong. Ive done every opiate under the sun. Tianeptine withdrawal is like coming off of fent, xanax, and ssri's all at once	100
Because there's too much money to be made with alcohol. I went through withdrawals and had to detox off of alcohol and benzos. It was miserable	100
Cymbalta withdrawal was the worst thing I've gone through. Months of pure hell.	100
8 people died and my son died the same week in the same area to black market fentanyl laced zanax. He had been trying to get help for insomnia and adhd. He worked for CCC of Ca found in gov bunk dead.	101
I've had 3 good friends die from shit laced with fentanyl. And a dozen more other ppl from my town I know who died from it. <*emoji*><*emoji*>	101
How do I go about getting involved with this??? My baby dad died in 2021 to an accidental OD, pills laced with fentanyl<*emoji*>	101
Cancer is a disease you can't choose it addiction is a choice I'm a addict and chose to awaken the beast now I choose to stay clean just saying	100
18 years clean. you got to want to stay clean	100
20 year meth addict here...never attempted to get sober before...I'm 10 months sober today<*emoji*>	100
You should be proud! That's an amazing accomplishment and congrats! You're an amazing warrior! Keep Going! <*emoji*>	010
What an amazing beautiful transformation Im so proud of you & your testimony <*emoji*>	010
Your son did not mean to, "overdose", and die. He was murdered by fentanyl laced drugs!!!!!	011
Grief is a very tricky and painful process, maybe just say I'm sorry for your loss?	010
healing love sent ur way babe and omg I'm so dam proud of you keep shinning!! your so brave so strong<*emoji*>	010
Wow this is devastating. I'm so sorry Lauri for your tremendous loss. No matter what you were going through, you always stood by your Son. May God bless you and your family thruthis difficult time. <*emoji*>	010
what a handsome young man, I'm so very sorry for your loss <*emoji*>	010
you might want to check dosing, you may be on too high of a dose or vice versa im only sleepy when I'm on too high of a dose and I'm 4 years in	110
they have been giving small doses of it to women when they give birth since forever	001
Tylenol taken daily or at high doses can cause issues too <*emoji*> they don't give education when they recommend it either	001

You should REALLY rethink taking more. Xanax is probably one of the top two worst withdrawals! People can die from seizures. Alcohol and Xanax are our most difficult withdrawals in the hospital, +Fent	011
absolutely heartbreaking, it needs to stop . my prayers for all the families dealing with a loss due to fentynal fuk fentynal	001
Accidental fentanyl overdose. He thought it was a Vicodin his friend gave him. It killed him instantly<*emoji*> Thank you friend	001
No, a lot of people still think that heroin or other drugs are "laced" with fentanyl. When they're JUST fentanyl. Fentanyl has replaced them.	001
I knew a girl that used to bully me that died from an accidental fentanyl overdose. Even though she caused me a lot of trauma, I was still very sad about her passing. She didn't deserve that.	001
Rehabs will help if they want to be clean. They have to want to be clean to stay clean.	001
THERE'S SUCH A THING AS LEGALLY PRESCRIBED OPIOIDS. BUT PPL RARELY 'RARELY OVERDOSE OR LEGALLY RX'D OPIOIDS UNDER A DR'S CARE'IF TAKEN CORRECTLY. ONLY OPIOID NAIVE PPL MIGHT OVERDOSE. BE RESPONSIBLE	001
pts w/ severe pain are suffering needlessly&killing themselves bc ppl think they need pain meds for a good time more-this is trend is NOT it	001

REFERENCE

1. Landis, J. R. & Koch, G. G. The measurement of observer agreement for categorical data. *Biometrics* **33**, 159–174 (1977).
2. Carpenter, K. A. *et al.* Which social media platforms facilitate monitoring the opioid crisis? *PLOS Digit. Health* **4**, e0000842 (2025).
3. Regulated Goods and Commercial Activities. https://www.tiktok.com/community-guidelines/en/regulated-commercial-activities.
4. Steen, E., Yurechko, K. & Klug, D. You Can (Not) Say What You Want: Using Algospeak to Contest and Evade Algorithmic Content Moderation on TikTok. *Soc. Media Soc.* **9**, (2023).
